# Short‐term effects of weight‐loading on heart rate variability in obese adults

**DOI:** 10.14814/phy2.70917

**Published:** 2026-05-21

**Authors:** Jakob Bellman, Per‐Anders Jansson, John‐Olov Jansson, Claes Ohlsson, Lennart Bergfeldt

**Affiliations:** ^1^ Department of Physiology, Institute of Neuroscience and Physiology Sahlgrenska Academy, University of Gothenburg Gothenburg Sweden; ^2^ Region Västra Götaland Sahlgrenska University Hospital Gothenburg Sweden; ^3^ Wallenberg Laboratory, Department of Molecular and Clinical Medicine, Institute of Medicine Sahlgrenska Academy, University of Gothenburg Gothenburg Sweden; ^4^ Sahlgrenska Osteoporosis Centre, Centre for Bone and Arthritis Research, Institute of Medicine Sahlgrenska Academy, University of Gothenburg Gothenburg Sweden; ^5^ Unit of Clinical Pharmacology, Department of Pharmaceuticals Sahlgrenska University Hospital Gothenburg Region Västra Götaland Sweden

**Keywords:** autonomic modulation, autonomic nervous system, heart rate variability, obesity, physical activity, weight‐loading

## Abstract

Weight‐loading reduces body fat, but its effects on cardiac autonomic modulation remain unclear. We examined heart rate variability (HRV) during free‐living weight‐loading in adults with class I obesity. Fifty‐one participants (27 females) were randomized to wear a heavy (11% of body weight) or a light vest (1%) for 8 h/day for 15 days. Twenty‐four‐hour ECG recordings were obtained before and on day 15. Heart rate (HR), relative HR reserve (%HRR), and time‐ and frequency‐domain HRV indices were analyzed across four predefined periods: morning (rest), afternoon (vest‐usage), evening, and night. During the afternoon period, the high versus low load increased HR (*p* = 0.0496) and %HRR (*p* = 0.03), while reducing SDNN (standard deviation of normal‐to‐normal intervals; *p* = 0.01) and RMSSD (root mean square of successive differences; *p* = 0.002). In females, but not in males, high load was associated with lower high‐frequency power (P_HF_), higher low‐frequency power (P_LF_), and an increased P_LF_/P_HF_ ratio during the afternoon, consistent with reduced parasympathetic modulation. No differences between groups were observed during resting periods. These findings indicate that weight‐loading increases cardiovascular workload and transiently alters autonomic modulation during active vest use, with more consistent responses in females and no sustained resting adaptations.

## 1 INTRODUCTION

Obesity is characterized by excessive body fat accumulation, defined as a body mass index (BMI) ≥30 kg/m^2^, and a significant public health issue. It is a major risk factor for various noncommunicable diseases across multiple organ systems (Schulze & Stefan, 2024; World Health Organization, 2024). With its global prevalence continuing to rise, understanding the physiological mechanisms linking excess adiposity to cardiovascular dysfunction has become a research priority (Magkos et al., 2024; Phelps et al., 2024; Speakman & Elmquist, 2022).

One proposed link between obesity and adverse cardiometabolic outcomes is dysregulation of the autonomic nervous system (ANS) (Guarino et al., 2017; Oliveira et al., 2020). The ANS, through its parasympathetic and sympathetic limbs, regulates various physiological processes, including heart rate (HR) and its variability (HRV), blood pressure, and energy expenditure (Hall & Hall, 2020). HRV analysis in the time and frequency domains provides non‐invasive measures of beat‐to‐beat variations in cardiac cycle intervals reflecting ANS influence on the sinus node. HRV is thereby regarded as marker of cardiac autonomic modulation, stress responses and overall cardiovascular health (Malik et al., 1996; Shaffer & Ginsberg, 2017; Souza et al., 2021; Thayer & Lane, 2009). Higher resting HRV is associated with improved cardiometabolic health. In contrast, lower resting HRV is associated with obesity, metabolic syndrome, and cardiovascular disease (Franz et al., 2013; Kiviniemi et al., 2017; Russo et al., 2021; Souza et al., 2021; Strüven et al., 2021). Furthermore, HRV indices are often interpreted as markers of autonomic tone or sympathovagal balance despite important methodological limitations (Bootsma et al., 2003; Eckberg, 1997; Tiwari et al., 2021). A more conservative view is that several HRV measures primarily reflect parasympathetic modulation, whereas sympathetic influences are not easily evaluated using HRV analysis.

Physical activity improves cardiometabolic health, for example by increasing insulin sensitivity and reducing waist circumference (Armstrong et al., 2022; Garthwaite et al., 2022; Qiu et al., 2023). Over time, regular exercise enhances resting parasympathetic modulation and increases HRV, despite acute reductions in HRV during periods of increased cardiovascular demand (Aagaard et al., 2014; Aubert et al., 2003; Kaikkonen et al., 2014). Even low‐intensity weight‐bearing activities, such as standing, have been associated with improved insulin sensitivity, higher energy expenditure, and reductions in waist circumference (Garthwaite et al., 2021; Shuval et al., 2015). Likewise, incorporating external weight‐loading, for instance through weighted vests, has been shown to promote fat loss and augment local energy expenditure, even without increases in structured physical activity (Bellman et al., 2024, 2025; Betts et al., 2019; Jansson et al., 2018; Ohlsson et al., 2020).

A weight‐loading homeostatic system sensing skeletal loading and regulating adiposity has previously been proposed (Jansson et al., 2018). Supporting this, the ATLAS trial demonstrated that externally applied weight‐loading reduced fat mass and increased lean mass over 35 days in adults with obesity (Bellman et al., 2025). However, the endogenous mechanical load imposed by excess adiposity in obesity does not appear to activate a corresponding compensatory reduction in fat mass. This suggests that chronic loading in obesity may dysregulate this homeostatic mechanism through pathways that remain to be identified. These observations raised the question of whether the ANS may serve as a mediator of loading‐dependent physiological responses. Weight‐loading, as an exercise‐like mechanical stimulus, increases metabolic demand and cardiovascular workload (Bellman et al., 2024), conditions known to attenuate parasympathetic modulation during periods of physical activity (Brockmann & Hunt, 2023). However, weight‐loading's short‐term effects on autonomic modulation of HRV under free‐living conditions have not been established. Whether ANS responses to loading differ between females and males also remains unknown. The HRV assessment was in the present sub‐study prespecified at Day 15, prior to the expected timeframe of meaningful body composition change. We hypothesized that higher versus lower weight‐loading would be associated with changes in HRV consistent with reduced parasympathetic modulation during periods of active vest use.

## 2 MATERIALS AND METHODS

### 2.1 Study design

The ATLAS trial was a single center randomized controlled trial conducted at Gothia Forum, Sahlgrenska University Hospital, Gothenburg, Sweden, from 2021 to 2022. Its primary aim was to evaluate the impact of weight‐loading on body weight, fat mass, and lean mass in individuals with obesity (Bellman et al., 2025). The present observational sub‐study investigated the effects of weight‐loading on HRV parameters reflecting ANS influence on the sinus node beating variability.

**FIGURE 1:**
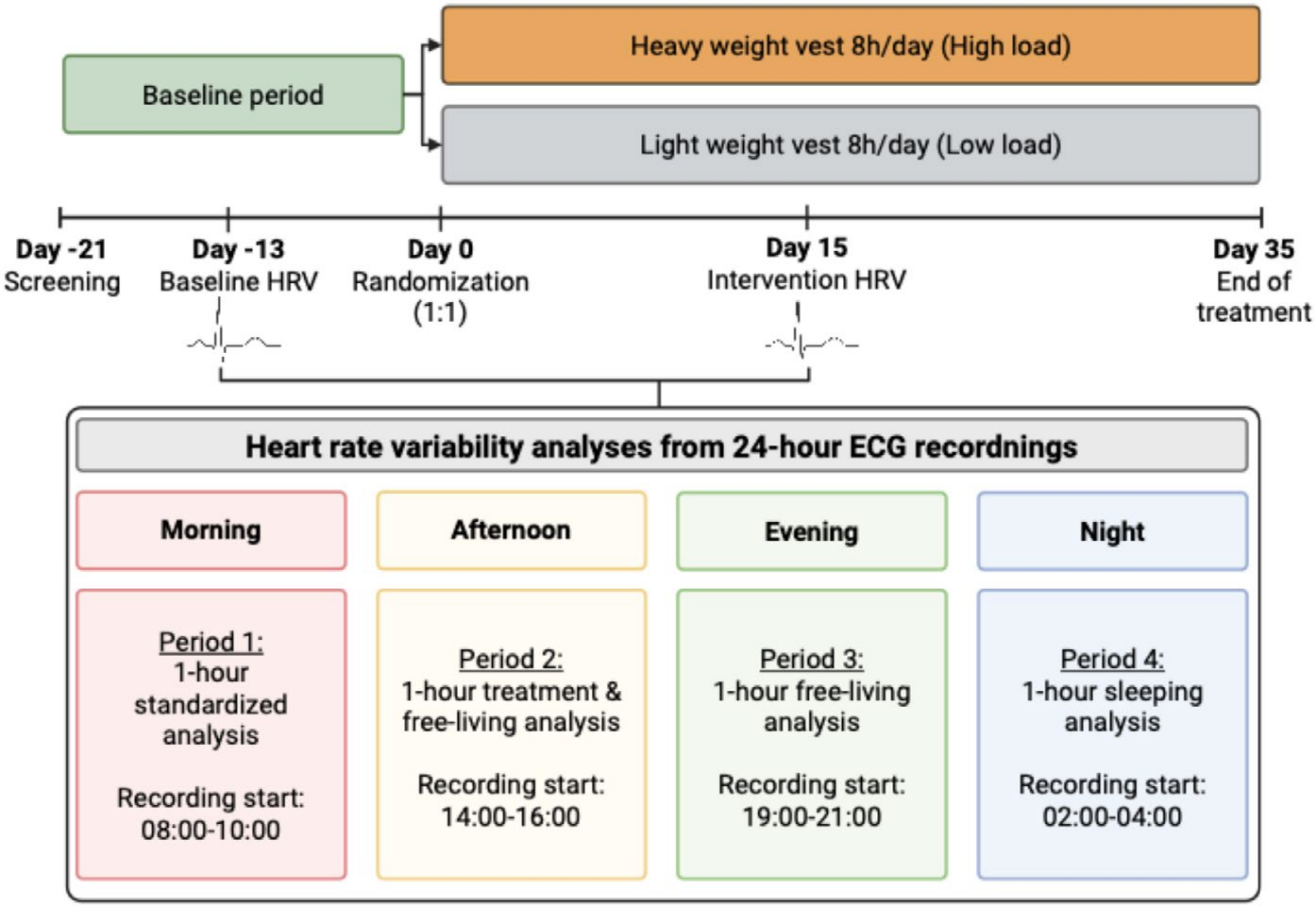
Schematic overview of the trial and HRV measurements timeline. Screening performed on Day −21. Baseline 24‐h ECG at Day −13 (*Baseline HRV*). Randomization on Day 0 to either Low‐load (*n* = 26) or High‐load (*n* = 25) vest use for 35 days. A second 24‐h ECG was obtained on Day 15 (*Intervention HRV*). HRV was analyzed over the full 24‐h period and during the four 1‐h periods (morning, afternoon, evening, night). Figure created in BioRender.com with publication rights. ECG, electrocardiography; HRV, Heart Rate Variability.

The study adhered to CONSORT (Consolidated Standards of Reporting Trials) guidelines. A schematic overview of the study is shown in Figure 1. It was approved by the Swedish Ethical Review Authority (approval number 2021‐00095) and conducted according to the Declaration of Helsinki and ICH‐GCP (International Conference on Harmonization Guidelines; Good Clinical Practice) standards. It is registered at ClinicalTrials.gov (NCT04697238) and an independent monitor oversaw the trial ensuring compliance and data integrity.

### 2.2 Participants

Adults aged 18–65 years with obesity class I (body mass index [BMI] 30–35 kg/m^2^) were enrolled. Exclusion criteria were diabetes, cardiovascular disease, previous bariatric surgery, impaired mobility, or chronic pain. The inclusion and exclusion criteria have been described in detail in the primary study publication and are shown in the study protocol (File S2) (Bellman et al., 2025). Self‐reported sex (male/female) was recorded during screening. All participants provided written informed consent prior to inclusion in the study. Concomitant medications were recorded throughout the trial and are reported in detail in a separate ATLAS publication (Bellman et al., 2025). No female participant was taking hormonal contraceptives during the study period.

### 2.3 Randomization and intervention

Out of 483 pre‐screened individuals, 112 were assessed for eligibility at screening, and 59 were randomized. A CONSORT flow diagram further illustrating study population has previously been published (Bellman et al., 2025). Participants were randomized in a 1:1 ratio to either:

1. High‐load group (HL): weight vest loaded to 11% of the body weight.
2. Low‐load group (LL): weight vest loaded to 1% of the body weight.

The weight vest (PRF Weight vest, Casall, Norrköping, Sweden) was identical except for the loading difference. The 10% difference in loading between groups was chosen to maximize intervention contrast while remaining within the vest's loading capacity. Randomization occurred on Day 0. The participants were instructed to wear the vests for a minimum of 8 h daily and remain standing for at least two of those hours. The intervention lasted 5 weeks. Compliance was monitored via diaries recording daily vest usage and standing time. Participants maintained their regular lifestyle outside the intervention. Blinding was not possible due to the nature of the intervention. Adverse events were documented throughout the study. All study restrictions are detailed in the study protocol (File S2).

### 2.4 Electrocardiography (ECG) recordings and HRV analysis

#### 2.4.1 ECG data collection

HR and HRV were assessed from 3‐channel 24‐h ECG recordings sampled at 256 Hz (Holter Recorder, LifeCard CF, Spacelabs Healthcare, UK) at baseline (Day −13) and on Day 15 during the intervention period. The second HRV measurement was chosen in the middle of the 35‐day weight vest intervention. The reason was that we wanted to explore any changes in the ANS preceding and possibly affecting or mediating intervention induced effects on body anthropometrics rather than being the result of them.

#### 2.4.2 HRV measurement periods

Continuous 24‐h ECG recordings were obtained from all participants. HRV was analyzed across the full 24‐h period as well as during four specific 1‐h sampling periods, selected to represent different physiological states and circadian ANS phases throughout the day. Each 1‐h period was selected within a predefined time window:

- *Period 1*, morning (08:00–10:00): Standardized resting measurement performed in a quiet supine setting, without the vest, following an overnight fast. This period was chosen for assessment of heart‐related variables at rest, not directly related to active vest wearing but presumably reflecting any carry‐over effects from the 15‐day intervention.
- *Period 2,* afternoon (14:00–16:00**):** Free‐living measurement during active vest use representing the intervention period. Vest use during this period was confirmed by the participant's diary records, making this the primary period for evaluating effects directly attributable to wearing the vest.
- *Period 3*, evening (19:00–21:00): Free‐living period without the vest. This period was selected to allow assessment of any carry‐over effects following active vest use in Period 2.
- *Period 4*, night (02:00–04:00): Sleep period without the vest, selected based on the participant's sleep diaries. This period was included to represent nocturnal autonomic modulation and allow assessment of any carry‐over effects of daytime loading.

##### Baseline assessment

Cardiac ANS modulation exhibits circadian variations, with a relative shift toward lower parasympathetic modulation during daytime hours (Li et al., 2011; Sammito et al., 2016). The baseline ECG recording at Day −13 was performed prior to randomization and the start of the intervention; no vest was worn during this recording. The baseline recording was used to explore how circadian variations influenced HRV indices in our study group and for internal validation of the methodology.

##### Intervention effects

The focus was to explore differences in intervention induced changes between baseline and intervention between the two loading groups for any of the 4 sampling periods and for females and males separately.

#### 2.4.3 ECG processing and data quality control

The ECG recordings were first processed automatically and then scrutinized manually using Pathfinder SL software (Spacelabs Healthcare, UK). During preprocessing, heartbeats were automatically detected from the ECG, and R‐R intervals were calculated. These intervals were then manually reviewed to correct errors caused by falsely detected beats. Any R‐R intervals due to non‐normal beats were replaced by cubic spline interpolation, thereby generating evenly sampled data.

For a sample period to be included in the analysis, 75% of the beats had to be normal sinus beats and, consequently, no more than 25% of non‐normal beats were accepted to be interpolated, following international recommendations (Malik et al., 1996). For each Period 1–4, a single 1‐h segment was selected for analysis. For Period 1, the timing was determined by logistical scheduling of the standardized study‐center visit. For Periods 2–4, candidate 1‐h segments were first required to fulfill predefined criteria (i.e., active vest use during Period 2, free‐living during Period 3, and sleep without vest use during Period 4). When these criteria were met, the 1‐h segment for analysis was selected based on ECG signal quality, prioritizing the segment with the lowest proportion of interpolated beats. Frequency‐domain parameters were calculated from the average of 12 consecutive 5‐min segments within each 1‐h period. The use of 5‐min segments for frequency‐domain analysis is in accordance with established guidelines for short‐term HRV analysis (Malik et al., 1996; Shaffer & Ginsberg, 2017). Averaging of all 12 consecutive segments within each hour was chosen to strengthen the representativeness of the HRV estimate over the full measurement period, rather than relying on a single 5‐min segment. If three or more 5‐min segments failed to meet the data quality criterion (proportion of normal beats), the entire 1‐h period was excluded from analysis. If two or fewer segments failed to meet this criterion, only those segments were omitted.

#### 2.4.4 Heart rate and HRV indices

Ten measures were derived: (1) HR, (2) HR reserve (HRR), (3) relative HRR (%HRR), (4) HRV triangular index (HRVI), (5) square root of the mean squared difference of successive NN‐intervals (RMSSD), (6) standard deviation of the average NN‐interval (SDANN), (7) standard deviation of NN‐interval (SDNN), (8) total spectral power (P_Total_), (9) high‐frequency power (P_HF_), and (10) low‐frequency power (P_LF_), the two latter were also normalized to the total power.

##### HRV—Time domain analysis

Time‐domain parameters were assessed for each 1‐h sample and for the full 24‐h recording to characterize overall HRV. RMSSD primarily reflects short‐term parasympathetic modulation, whereas SDNN and the HRVI provide estimates of overall HRV. SDANN reflects longer‐term components of HRV related to slower fluctuations across the recording period (Malik et al., 1996; Shaffer & Ginsberg, 2017). HRVI was calculated as the integral of the NN interval histogram divided by the maximum height of the histogram (Malik et al., 1996).

##### HRV—Frequency domain analysis

Power spectrum analysis was employed using Fast Fourier transformation (FFT) based on Welch's periodogram method providing information of how HRV varies within different frequency ranges. Variance or power was measured in milliseconds squared (ms^2^) and distributed according to frequency (Hz). Parameters included P_Total_, P_VLF_ (0.017–0.04 Hz), P_LF_ (0.04–0.15 Hz), and P_HF_ (0.15–0.40 Hz). P_HF_ primarily reflects parasympathetic modulation of heart rate. P_LF_ reflects a combination of parasympathetic and sympathetic influences and cannot be interpreted as a pure measure of sympathetic modulation. The P_LF_/P_HF_ ratio is reported for completeness and comparability with existing literature, but should not be regarded as a reliable index of sympathovagal balance (Billman, 2013; Malik et al., 1996; Shaffer & Ginsberg, 2017). P_HF_ and P_LF_ were also evaluated in normalized units (n.u.), representing the relative value of each power component in proportion to the total power minus the P_VLF_ component. The role of P_VLF_ in autonomic regulation is not fully understood but lower P_VLF_ has been associated with increased mortality in for example cardiovascular disease (Hadase et al., 2004; Shaffer & Ginsberg, 2017). Utilizing normalized values allows for the assessment of low and high‐frequency components, independently of variations in total spectral power (Berntson et al., 1997; Malik et al., 1996; Shaffer & Ginsberg, 2017).

#### 2.4.5 Heart rate, work‐load estimate, and blood pressure

This part of the protocol was included to provide a physiological background for the HRV analysis. HR is a strong determinant for HRV and was estimated for all time periods and for the entire 24‐h recordings. HR reserve (HRR) and %HRR were calculated to estimate cardiovascular workload during the afternoon period with physical activity and weight‐loading compared to the resting morning period. HRR was calculated as the maximum HR minus resting HR (Karvonen et al. 1957). Maximum HR was either estimated as 220 minus age or the highest HR recorded during Day −13 and Day 15, whichever was highest (Fox & Naughton, 1972). Resting HR was the mean value from the standardized 1‐h morning period. %HRR was calculated as the percentage of HRR utilized during in the afternoon period: %HRR = [(HR_weight‐loading_–HR_rest_)/(HR_max_–HR_rest_)] × 100, in accordance with previous publications (Aagaard et al., 2014; Karvonen et al., 1957).

Blood pressure was measured on Day 0 and Day 15 using a Dinamap Carescape Vital Signs Monitor (GE Healthcare, Illinois, USA) after at least 5 min of rest in a supine position.

### 2.5 Outcome measures and statistical analysis

Outcome variables were changes between baseline (Day −13) and intervention (Day 15) for the HRV time and frequency domain indices, computed independently for each predefined time segment. Additional outcome variables were changes in HR, %HRR, systolic and diastolic blood pressure. Results were compared between the HL and LL groups as absolute and percentage changes from baseline and for females and males separately.

Descriptive data were analyzed following per protocol principles, requiring participants to adhere to the study protocol (see Additional File S2). All 51 eligible participants were included in the analysis, and no imputation was performed despite a few missing data points. Since endpoints were exploratory, statistical analyses were not adjusted for multiplicity. All continuous variables were assessed for normality using the Shapiro–Wilk test. Many HRV variables showed significant deviation from normality, and non‐parametric methods were therefore used throughout as a conservative analytical approach to obtain as robust results as possible. Results are presented as medians and interquartile ranges (IQR). Between‐group differences were assessed using Mann–Whitney's U test and within‐group differences using Wilcoxon's signed‐rank test. Outliers were assessed by visual inspection of box plots and are displayed in figures. Statistical analyses and figures were created using SPSS Statistics 29 (IBM Corp., Armonk, NY, USA), GraphPad Prism 10 (GraphPad Software, Boston, MA, USA) or BioRender (Science Suite Inc., Toronto, Ontario, Canada). A *p* < 0.05 was considered statistically significant.

## 3 RESULTS

### 3.1 Study participants

A total of 51 participants (26 in the LL and 25 in the HL group) were included in the analysis, comprising 27 females and 24 males. Their characteristics are presented in Table 1. The groups were well balanced for age and sex. Overall, 98% of the beats were normal and included in the analysis. However, one participant in the LL group was excluded from the frequency‐domain analysis in the second measurement (Day 15) due to excessive non‐normal heartbeats. In the HL group, exclusions of specific time periods were made for the same reason. For time‐domain analyses, two participants were excluded from the morning and afternoon periods, and five from the evening and night periods. Similarly, for frequency‐domain analyses, one participant was excluded from the morning, five from the afternoon, four from the evening, and five from the night period. The final number of participants included in each analysis are presented in Table 1. Figures in the main manuscript illustrate the primary findings of the study, whereas detailed numerical values for all outcome variables, including absolute and relative changes from baseline and sex‐stratified analyses, are provided in the supplementary File S1 (Tables S1–S5).

**TABLE 1:** Baseline characteristics.

Characteristics	Low load			High load			*p* (Mann–Whitney *U*)
	Median	IQR	*n*	Median	IQR	*n*	
Age (years)	44.0	16.0	26	45.0	16.0	25	NS
Sex, females, *n* (%)	13 (50%)		26	14 (56%)		25	NS
Height (cm)	180.0	38.0	26	174.0	8.0	25	NS
Weight (kg)	104.4	18.9	26	99.9	9.6	25	NS
BMI (kg/m^2^)	32.5	3.2	26	33.2	2.0	25	NS
Beats qualified, 24‐h (*n*)	109,664	17,739	26	110,647	30,023	25	NS
Beats qualified (%)	99.7	0.6	26	99.9	1.0	25	NS
HR, 24 h (bpm)	75	11	26	79	14	25	NS
HR, Morning (bpm)	69	15	26	74	14	23	NS
HR, Afternoon (bpm)	78	12	26	79	22	23	NS
HR, Evening (bpm)	77	17	26	80	21	20	NS
HR, Night (bpm)	62	7	26	66	13	20	NS
HRR (bpm)	106	19	26	104	20	23	NS
Relative HRR (%HRR)	9.2%	7.5%	26	3.9%	13.5%	23	NS
Systolic Blood Pressure (mmHg)	120	12	26	122	13	25	NS
Diastolic Blood Pressure (mmHg)	75	16	26	71	15	25	NS
Time domain (ms)							
SDNN, 24 h	142	35	26	138	50	23	NS
SDNN, Morning	98	55	26	91	41	23	NS
SDNN, Afternoon	69	34	26	69	33	23	NS
SDNN, Evening	65	32	26	68	39	20	NS
SDNN, Night	79	36	26	72	35	20	NS
RMSSD, 24 h	29	12	26	27	11	23	NS
RMSSD, Morning	28	21	26	26	22	23	NS
RMSSD, Afternoon	22	10	26	27	17	23	NS
RMSSD, Evening	21	10	26	22	11	20	NS
RMSSD, Night	32	14	26	35	25	20	NS
Frequency domain (ms^2^)							
P_Total_, morning	1641	2198	25	2176	1928	24	NS
P_Total_, afternoon	1159	1028	25	1397	1737	20	NS
P_Total_, evening	1241	1991	25	1274	1290	21	NS
P_Total_, night	1846	2157	25	2178	1827	20	NS
Frequency domain							
Normalized values (n.u.; ms^2^)							
P_HF_ n.u., morning	34	16	25	33	20	24	NS
P_HF_ n.u., afternoon	17	10	25	19	13	20	NS
P_HF_ n.u., evening	20	16	25	20	23	21	NS
P_HF_ n.u., night	40	28	25	32	31	20	NS
P_LF_ n.u., morning	63	18	25	66	24	24	NS
P_LF_ n.u., afternoon	81	12	25	80	15	20	NS
P_LF_ n.u., evening	76	19	25	78	27	21	NS
P_LF_ n.u., night	57	26	25	65	32	20	NS
Ratio P_LF_/P_HF_ n.u., morning	1.9	1.4	25	2.0	2.5	24	NS
Ratio P_LF_/P_HF_ n.u., afternoon	4.6	3.7	25	4.1	4.2	20	NS
Ratio P_LF_/P_HF_ n.u., evening	3.6	4.1	25	3.9	5.0	21	NS
Ratio P_LF_/P_HF_ n.u., night	1.4	1.7	25	2.1	2.5	20	NS

Abbreviations: BPM, beats per minute; ECG, electrocardiography; HR, heart rate; HRR, heart rate reserve; Relative HRR (%HRR), percentage of HRR used during weight‐loading; NN, interval between normal ECG R‐peaks; NS, non‐significant; n.u., normalized units; P_HF_, power in high frequency; P_LF_, power in low frequency; P_Total_, total power spectral density; RMSSD, Square root of the mean squared difference of successive NN‐intervals; SDANN, Standard deviation of the average NN‐interval; SDNN, Standard deviation of NN‐interval; Triangular index, integral of density distribution.

Overall, there were substantial inter‐individual differences and wide ranges of variation. The pattern of directional changes therefore became more important for the interpretation of the effects of the intervention than any individual statistically significant difference.

### 3.2 Baseline circadian variation

The circadian variations of HR, relative HRR, and HRV indices across different time periods at baseline corroborated previous observations and there were no significant differences between the HL and LL groups (Figures S1 and S2).

### 3.3 Effect of high versus low load on heart rate, workload, and blood pressure

**FIGURE 2:**
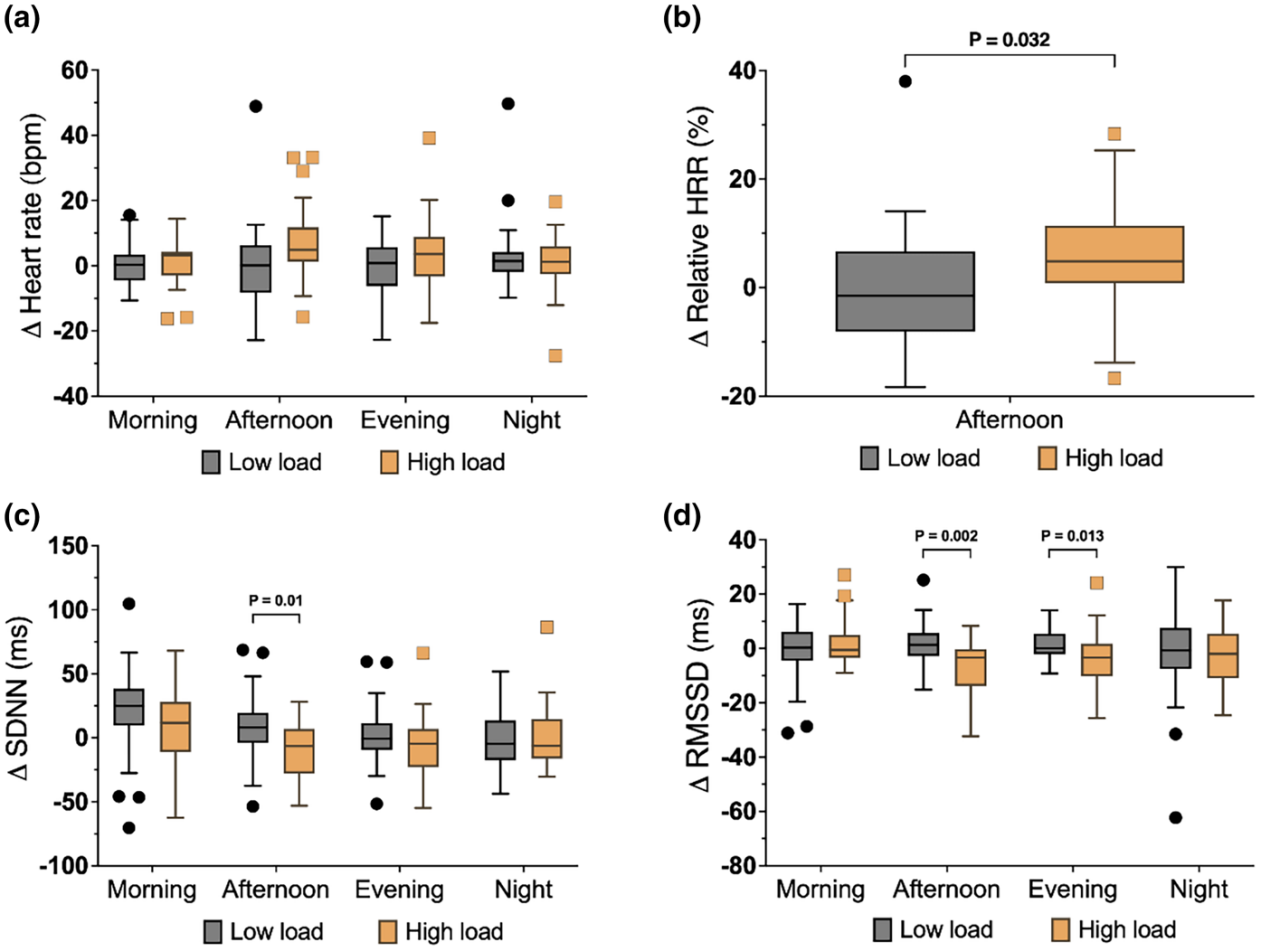
Changes in heart rate and time domain HRV parameters from baseline in the low‐load (*n* = 26) and high‐load (*n* = 20–23) groups. Panels show changes in (a) heart rate, (b) relative HRR (%HRR), (c) SDNN, and (d) RMSSD across four time periods (morning, afternoon, evening, night). The high‐load intervention increased %HRR and reduced SDNN and RMSSD during the afternoon. Box plots display medians (lines), interquartile ranges (boxes), whiskers (Tukey's method), and outliers (points). Abbreviations are provided as shown in Table 1.

During the afternoon period, the HL group had a ~7% higher HR (*p* = 0.0496; Table S3) and a ~6% higher relative HRR compared to the LL group (*p* = 0.03; Figure 2). These differences were expected and both confirmed the participants' adherence to the study protocol, and that HL was sufficiently different from LL.

No significant differences between the groups were observed for 24‐h HR, diastolic or systolic blood pressure, suggesting that the intervention did not lead to sustained cardiovascular changes.

### 3.4 Effect of high versus low load on HRV indices

#### 3.4.1 Time domain analyses

During the afternoon period, the HL group had significantly lower SDNN (*p* = 0.01) and RMSSD (*p* = 0.002) compared to the LL group (Figure 2). RMSSD remained lower in the HL group during the evening period (*p* = 0.013; Figure 2). No significant between‐group differences were observed for SDANN or HRVI.

#### 3.4.2 Frequency domain analyses

**FIGURE 3:**
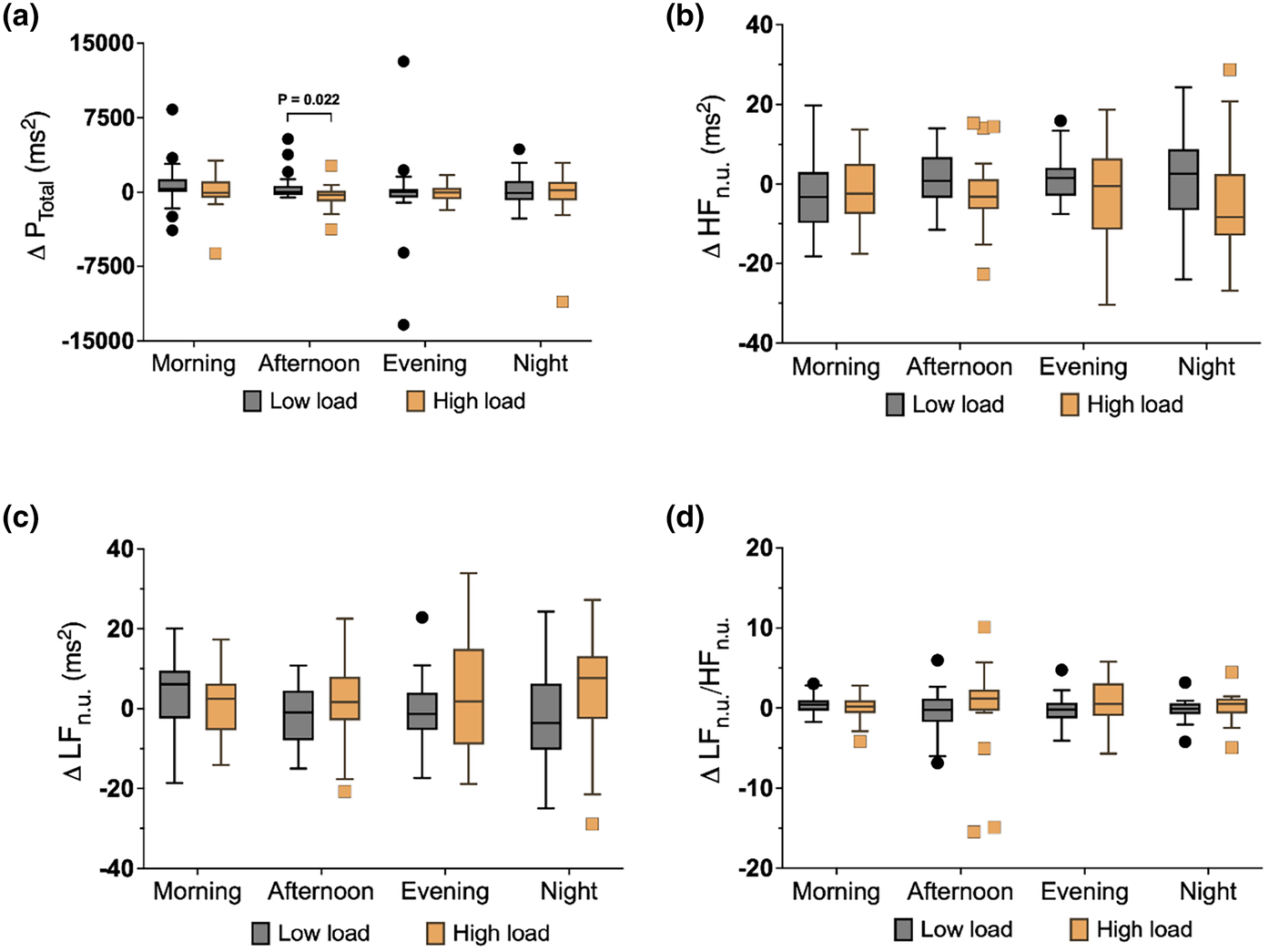
Changes in frequency domain HRV parameters from baseline in the low‐load (*n* = 25) and high‐load (*n* = 20–24) groups. Panels show changes in (a) total power spectral density (P_Total_), (b) normalized high‐frequency power (HF_n.u._), (c) normalized low‐frequency power (LF_n.u._), and (d) the LF_n.u._/HF_n.u._ ratio across four time periods (morning, afternoon, evening, night). Total power was significantly lower in the high‐load group during the afternoon. Box plots show medians (lines), interquartile ranges (boxes), whiskers (Tukey's method), and outliers (points). Abbreviations are provided as shown in Table 1.

In the HL group, total spectral power (P_Total_) decreased during the afternoon period compared to the LL group (*p* = 0.022; Figure 3). In the afternoon period, P_HF n.u._ decreased in the HL group, whereas P_LF n.u._ and the P_LF n.u._/P_HF n.u._ ratio increased numerically. However, none of these differences reached statistical significance between groups (Figure 3).

### 3.5 Sex‐stratified analysis of HRV responses

**FIGURE 4:**
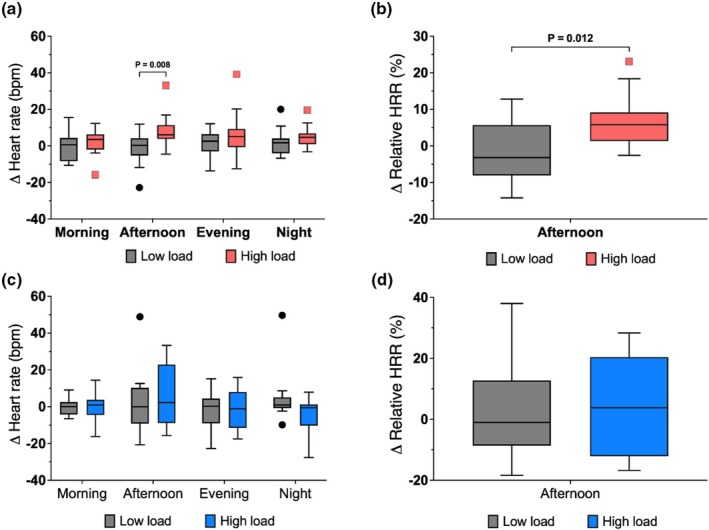
Changes in heart rate and %HRR from baseline in females and males. Panels show changes in heart rate (a, c) and %HRR (b, d) across four time periods (morning, afternoon, evening, night) in females (a, b; low‐load *n* = 13, high‐load *n* = 11–13) and males (c, d; low‐load *n* = 13, high‐load *n* = 9–11). Gray bars represent the low‐load group; red bars represent the high‐load group in females and blue bars represent the high‐load group in males. In females, the high‐load group showed increased afternoon heart rate and %HRR compared with low load. Box plots show medians (lines), interquartile ranges (boxes), whiskers (Tukey's method), and outliers (points). Abbreviations are provided as shown in Table 1.

Sex‐stratified analyses revealed different responses between females and males, which may help to explain the large inter‐individual variability observed in the overall cohort; Table 2 and Figure 4. The HL group comprised 14 females and 11 males and the LL group 13 females and 13 males. Visual inspection of IQR suggested that variability differed between the sexes for several parameters, where females generally exhibited narrower IQR for HR during the afternoon period than males. Overall, females showed more consistent changes in HRV indices across periods, while changes were more heterogeneous in males.

**TABLE 2:** Sex‐stratified changes between baseline and Day 15 in heart rate, cardiovascular workload and HRV responses during the afternoon period in the low‐ and high‐load groups.

	Females LL	Females HL	*p*‐value	Males LL	Males HL	*p*‐value
	Median [IQR]	Median [IQR]		Median [IQR]	Median [IQR]	
HR, (*bpm*)	0.3 [10]	**6** [8] **	**0.008**	0 [20]	2 [32]	0.693
%HRR (*%*)	−3% [14%]	**6%** [8%] **	**0.012**	‐1% [21%]	4% [33%]	0.483
RMSSD, (*ms*)	2 [10]	**−8** [12] *	**0.005**	0.4 [9]	−2 [20]	0.091
P_HF_ n.u., (*ms* ^*2*^)	−1 [12]	**−6** [9] **	**0.021**	3 [12]	1 [16]	0.771
P_LF_ n.u., (*ms* ^*2*^)	1 [11]	**8** [13] *	**0.049**	−4 [10]	−1 [16]	0.821
Ratio P_LF_/P_HF_ n.u.	0.3 [4]	**2** [4] **	**0.026**	−1 [4]	−0.2 [9]	0.674

*Note*: Abbreviations are provided as shown in Table 1. Bold *p*‐values indicate statistically significant between‐group differences (*p* < 0.05).

* Significant within‐group changes (*p* < 0.05).

** *p* < 0.01.

In females, HL versus LL was associated with a significant increase in HR and HRR% during the afternoon period (*p* = 0.012). No such differences were observed in males. For time domain HRV measures, females in the HL group showed a significant reduction in RMSSD during both the afternoon (*p* = 0.005) and evening periods (*p* = 0.021). In contrast, in males the HL group had lower SDNN than the LL group during the afternoon (*p* = 0.021; Table S5).

**FIGURE 5:**
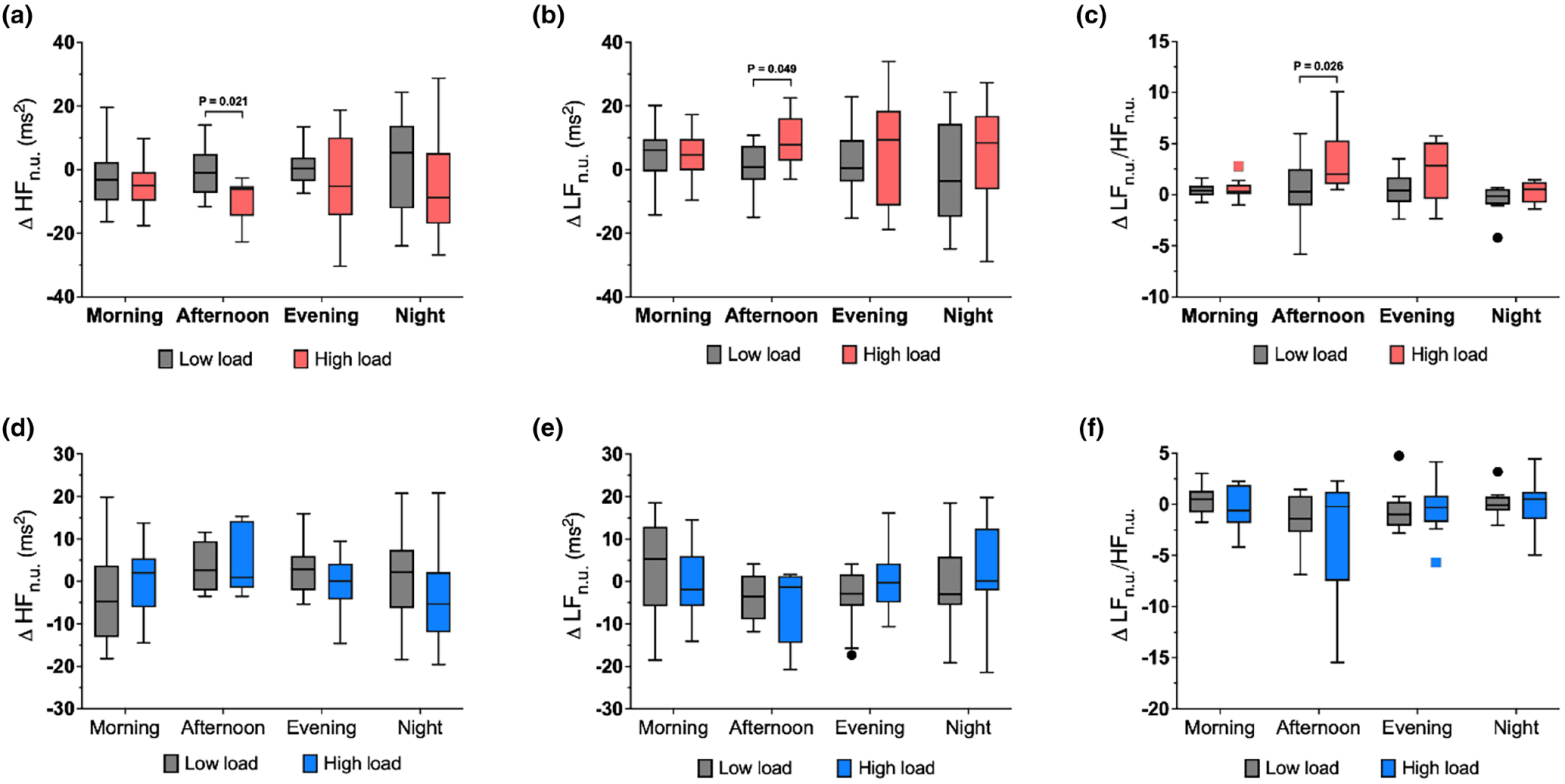
Changes in frequency domain HRV parameters from baseline in females and males. Panels show changes in normalized high‐frequency power (HF_n.u._), normalized low‐frequency power (LF_n.u._), and the LF_n.u._/HF_n.u._ ratio across four time periods (morning, afternoon, evening, night) in females (a–c; low‐load *n* = 13, high‐load *n* = 10–13) and males (d–f; low‐load *n* = 12, high‐load *n* = 9–11). Gray bars represent the low‐load group; red bars represent the high‐load group in females and blue bars represent the high‐load group in males. In females, the high‐load group showed reduced HF_n.u._, increased LF_n.u._, and a higher LF_n.u._/HF_n.u._ ratio in the afternoon compared with low load. Box plots show medians (lines), interquartile ranges (boxes), whiskers (Tukey's method), and outliers (points). Abbreviations are provided as shown in Table 1.

Frequency‐domain analyses further supported this pattern; Figure 5. In females, the HL group had a significant reduction in P_HF n.u._ (*p* = 0.021) along with an increase in P_LF n.u._ (*p* = 0.049) and the P_LF n.u._/P_HF n.u._ ratio (*p* = 0.026) during the afternoon period. Within the HL group the P_LF n.u._/P_HF n.u._ ratio remained elevated during the evening period, suggesting potential carry‐over effects (*p* < 0.05; Table S4). In contrast, males showed no significant differences between groups in any frequency‐domain parameter. Detailed numerical values for sex‐stratified analyses are provided in Tables S4 and S5.

## 4 DISCUSSION

This study explored how transient weight‐loading affects HRV indices as indicators of ANS modulation of the sinus node and its firing variability in individuals with obesity. Carrying 11 vs. 1% of body weight during 8 h per day for 15 days increased cardiovascular workload and was associated with reduced indices of parasympathetic modulation (lower RMSSD and P_HF_), alongside patterns consistent with a shift in autonomic modulation toward lower‐frequency components. These effects were more consistently observed in females and also suggested an acute carry‐over effect into the evening period, when most participants were no longer wearing the vest. However, no lasting autonomic or cardiovascular adaptations were observed, as HRV remained unchanged between the two morning resting periods. Together, these findings suggest that weight‐loading induces acute alterations in autonomic modulation and cardiovascular function during active loading. Some transient effects may persist shortly after vest removal, but no sustained changes in resting autonomic modulation were observed over the study period.

### 4.1 Impact of weight‐loading on cardiovascular workload

HR and %HRR increased in the HL group during active free‐living vest wearing, reflecting a greater cardiovascular workload. Previous studies have examined HR responses to weight vests in controlled settings, such as comparing standing with a vest to sitting (Bellman et al., 2024). To our knowledge, the effects of prolonged weight‐loading in a free‐living setting on cardiovascular function have not been investigated. Overall, our findings align with established physiological responses to physical activity, including increased peripheral metabolic demand and elevated cardiac output (Ashcroft et al., 2024; Michael et al., 2017). Our results suggest that weight‐loading can increase cardiovascular workload even in the absence of structured exercise. Specifically, the larger increases in HR and %HRR observed in females suggest a higher relative physiological strain during weight‐loading, which may have elicited more uniform autonomic responses detectable by HRV analysis.

### 4.2 ANS responses to weight‐loading

In this study, analyses showed reduced HRV (lower SDNN, RMSSD and P_HF n.u._) corresponding to reduced parasympathetic modulation during HL weight‐loading. In addition, RMSSD remained reduced in the evening, suggesting a short‐term carry‐over effect with reduced parasympathetic modulation after vest removal. Numerical changes in P_LF n.u._ and the P_LF n.u._/P_HF n.u._ ratio were also observed. As pointed out in Methods, these indices are often interpreted as reflecting increased sympathetic contributions; however, their physiological interpretation remains debated, and they should not be directly interpreted as measures of sympathovagal balance (Billman, 2013; Bootsma et al., 2003; Eckberg, 1997). They are reported herein for completeness and to facilitate comparison with the broader HRV literature.

Individuals with obesity frequently exhibit chronically reduced HRV, consistent with lower parasympathetic modulation and altered ANS modulation toward lower‐frequency components (Grassi et al., 2019; Lambert et al., 2019). Such patterns have been associated with long‐term metabolic dysregulation and elevated cardiovascular risk (Bartness et al., 2009; Fisher et al., 2015; Smith & Minson, 2012). While sustained elevations in sympathetic influences on cardiovascular regulation are considered harmful, transient sympathetic activation—such as during physical activity—may promote beneficial physiological responses, including lipolysis, thermogenesis, and anti‐inflammatory effects. However, interventions specifically targeting ANS modulation in obesity remain poorly explored (Cohen & Kajimura, 2021; Saxton et al., 2019; Thorp & Schlaich, 2015; Wang et al., 2022).

Our findings resemble the acute response typically observed during exercise (Brockmann & Hunt, 2023; Souza et al., 2021). It is worth noting, however, that different forms of exercise exert different effects on ANS modulation. Dynamic aerobic exercise acutely suppresses parasympathetic modulation through cardiac acceleration and is associated with improved (increased) resting HRV following prolonged training (Aubert et al., 2003; Carter et al., 2003; Hautala et al., 2003). Resistance and isometric exercise similarly reduce HRV during and immediately following active exercise, reflecting decreased parasympathetic modulation, with more variable effects on resting autonomic function following prolonged training (Heffernan et al., 2007; Kingsley & Figueroa, 2016). Weight‐loading via a weighted vest represents a physiologically intermediate stimulus by increasing gravitational load and activating postural muscles. The observed reductions in RMSSD and P_HF_ during active vest use are consistent with the ANS response to increased mechanical and metabolic demand, reflecting transient parasympathetic withdrawal during periods of physical work. The absence of effects during resting periods suggests that the observed changes were primarily short‐term responses to active loading rather than sustained regulatory adaptations. The 15‐day intervention may, however, have been too short to induce lasting resting‐state adaptations in HRV indices.

### 4.3 Sex differences in HRV changes

Sex‐stratified analyses revealed more consistent HRV changes in females, particularly during and after active vest use. Although variance seemed to differ between females and males for some parameters, this did not fully explain the group differences observed. The group of males might be more heterogeneous than the group of females, where for example, differences in physical fitness among males may be one factor. Another factor might be larger variability in body composition within the group of males. On average, females have a lower proportion of lean body mass relative to total body weight compared to males (Liu et al., 2021). Since the weight of the vest was calculated based on total body weight rather than lean mass, females may have experienced a greater relative effort in response to weight‐loading. Furthermore, the vests were similar for males and females and not anatomically adjustable, which might have caused more discomfort among females. The underlying mechanisms behind these sex differences remain unclear but warrant further investigation.

Previous studies have reported sex differences in HRV measures, with females showing higher P_HF_ and RMSSD compared to males, despite higher resting HR (Antelmi et al., 2004; Koenig & Thayer, 2016). These findings have often been interpreted as reflecting greater parasympathetic influence in females. However, when sympathovagal balance was assessed using pharmacological blockade of the ANS, a more direct method than HRV analysis, no significant difference between females and males was found in sinoatrial node ANS modulation or intrinsic HR at rest (Bergfeldt & Vahedi, 2026). In the present interventional study, females displayed more consistent HRV responses, particularly reductions in RMSSD and P_HF n.u._, along with responses in P_LF n.u._ and the P_LF n.u._/P_HF n.u._ ratio indicative of altered autonomic modulation during weight‐loading. Notably, the physiological background to HRV is very complex and HRV measures do not allow for reliable direct quantification of sympathovagal balance (Bootsma et al., 2003; Eckberg, 1997). The more consistent HRV response observed in females in our study should therefore be interpreted cautiously regarding the influence of the two ANS limbs and considered hypothesis‐generating.

### 4.4 Methodological aspects and study limitations

Strengths of this study include its randomized design, adherence to ICH guidelines, and comprehensive evaluation of HRV indices across multiple time periods. The high quality of the 24‐h ECG recordings further ensured reliable data, with over 98% of heartbeats included in the analyses.

There are several limitations to this study. First, blinding was not possible due to the noticeable weight difference between vests. Second, vest usage (duration and activity) was self‐reported, which may introduce recall bias although daily diaries were kept. Third, although the participants wore accelerometers during the study period, these data were analyzed as weekly means in a separate publication and were not time‐synchronized with the HRV measurement windows, precluding objective characterization of physical activity during individual analysis periods (Bellman et al., 2025). Similarly, while sleep diaries guided selection of the nocturnal analysis window, objective verification of sleep was not performed. Fourth, measurement conditions were standardized with respect to timing and recording procedures, but respiratory rate and posture were not controlled, which limits physiological interpretation of HRV indices under free‐living conditions. Fifth, the intervention‐related HRV assessment was performed on Day 15, rather than at the end of the 35‐day weight‐loading intervention. This limits conclusions regarding longer‐term autonomic adaptations. A second measurement closer to Day 35 would have provided insight into sustained effects but was not performed due to logistical constraints. Sixth, no circulating biomarkers were collected during the afternoon measurement period, limiting our ability to relate HRV changes to concurrent neuroendocrine or cardiovascular responses.

In addition, the study population was restricted to individuals with class I obesity (BMI 30–34.9 kg/m^2^), which limits generalizability of the results to individuals with more severe obesity or no obesity at all (overweight or normal weight). Finally, this sub‐study was not powered or designed to evaluate sex‐specific effects on HRV, and HRV change was not a primary endpoint of the ATLAS trial. Hormonal profiling was not performed, and menstrual cycle phase was not recorded. HRV has been shown to vary across the menstrual cycle in association with fluctuations in ovarian hormones, although the relative contributions of estradiol and progesterone remain inconsistent across studies (Salih & Abdulateef, 2026; Schmalenberger et al., 2019, 2020). The participants in our study were assessed under free‐living conditions without standardization to a specific cycle phase. The females were therefore supposedly unlikely to have been synchronized with respect to the menstrual phase, and measurements presumably reflected a randomly distributed range of hormonal states, in the high versus low load groups and at baseline versus day 15. Whether any difference in hormonal states contributed to inter‐individual variability in HRV indices and affected our measures on the intervention effects in females can, however, not be ruled out. The observed sex‐related differences in HRV responses should be interpreted with caution and as hypothesis‐generating. Future studies should incorporate tighter control of physiological confounders (including menstrual cycle phase standardization or direct hormonal profiling in female participants and related changes in body temperature affecting HR/HRV), repeated measurements across longer intervention periods, and time‐specific biomarker sampling to better elucidate the mechanisms underlying temporal HRV responses to weight‐loading.

## 5 CONCLUSION

Wearing a high‐load weight vest increased cardiovascular workload and induced transient changes in HRV indices consistent with reduced parasympathetic modulation during active vest use. These effects were more pronounced in females, with some indications of short‐term carry‐over effects into the evening period. However, no sustained changes in resting autonomic or cardiovascular function were detected beyond periods of vest use. Thus, weight‐loading – like physical activity in general – appears to acutely influence ANS modulation and cardiovascular function, but its long‐term clinical implications remain uncertain. Future studies should evaluate whether weight‐loading interventions can support reductions in fat mass while preserving or increasing lean mass, and whether transient HRV responses translate into meaningful long‐term adaptations in ANS regulation. The potential integration of weight‐loading with pharmacological obesity treatments also warrants further investigation.

## AUTHOR CONTRIBUTIONS

**Jakob Bellman:** Conceptualization; data curation; formal analysis; funding acquisition; investigation; methodology; project administration; resources; software; validation; visualization. **Per‐Anders Jansson:** Conceptualization; funding acquisition; investigation; methodology; project administration; resources; software; supervision; validation; visualization. **John‐Olov Jansson:** Conceptualization; funding acquisition; investigation; methodology; project administration; resources; supervision; validation; visualization. **Claes Ohlsson:** Conceptualization; data curation; funding acquisition; investigation; methodology; project administration; resources; software; supervision; validation; visualization. **Lennart Bergfeldt:** Conceptualization; data curation; formal analysis; funding acquisition; investigation; methodology; project administration; resources; software; supervision; validation; visualization.

## FUNDING INFORMATION

The study was financed by grants from the Swedish state under the agreement between the Swedish government and the county councils, the ALF‐agreement (ALFGBG‐965051 and ALFGBG‐979083), Knut and Alice Wallenberg Foundation (KAW 2020.0230), the Torsten Söderberg Foundation (MT3/20), Göteborgs Läkaresällskap (The Gothenburg Society of Medicine; GLS‐1020343), Stiftelsen Längmanska kulturfonden (BA25‐2101), Stiftelsen fru Mary von Sydows, född Wijk, donationsfond (2024‐139), and Stiftelsen Lars Hiertas minne (FO2024‐0310). The funders of the study had no role in study design, data collection, data analysis, data interpretation, or writing of the report.

## CONFLICT OF INTEREST STATEMENT

The authors declare no conflicts of interest.

## ETHICS STATEMENT

The trial was approved by the Swedish Ethical Review Authority (registration number 2021–00095) and was conducted in accordance with the Declaration of Helsinki and the International Conference on Harmonization Guidelines (ICH) for Good Clinical Practice (GCP).

## CONSENT

All participants provided written informed consent before undertaking any study procedures.

## Supporting information


**Figure S1:** Baseline time domain values.
**Figure S2:** Baseline frequency domain values.
**Table S1:** Baseline characteristics for additional parameters not included in main manuscript.
**Table S2:** Absolute changes from baseline.
**Table S3:** Relative changes from baseline.
**Table S4:** Sex‐stratified analyses: Females absolute changes.
**Table S5:** Sex‐stratified analyses: Males absolute changes.

Data S2.

## Data Availability

Restrictions apply to the availability of the data generated or analyzed during this study to preserve patient confidentiality. The corresponding author will on request detail the restrictions and any conditions under which access to some data may be provided. Study protocol will be available with this publication.

## References

[phy270917-bib-0001] Aagaard, P. , Sahlén, A. , Bergfeldt, L. , & Braunschweig, F. (2014). Heart rate and its variability in response to running—Associations with troponin. Medicine and Science in Sports and Exercise, 46(8), 1624–1630. 10.1249/MSS.0000000000000270 24504429

[phy270917-bib-0002] Antelmi, I. , De Paula, R. S. , Shinzato, A. R. , Peres, C. A. , Mansur, A. J. , & Grupi, C. J. (2004). Influence of age, gender, body mass index, and functional capacity on heart rate variability in a cohort of subjects without heart disease. The American Journal of Cardiology, 93(3), 381–385. 10.1016/j.amjcard.2003.09.065 14759400

[phy270917-bib-0003] Armstrong, A. , Jungbluth Rodriguez, K. , Sabag, A. , Mavros, Y. , Parker, H. M. , Keating, S. E. , & Johnson, N. A. (2022). Effect of aerobic exercise on waist circumference in adults with overweight or obesity: A systematic review and meta‐analysis. Obesity Reviews, 23(8), e13446. 10.1111/obr.13446 35383401 PMC9540641

[phy270917-bib-0004] Ashcroft, S. P. , Stocks, B. , Egan, B. , & Zierath, J. R. (2024). Exercise induces tissue‐specific adaptations to enhance cardiometabolic health. Cell Metabolism, 36(2), 278–300. 10.1016/j.cmet.2023.12.008 38183980

[phy270917-bib-0005] Aubert, A. E. , Seps, B. , & Beckers, F. (2003). Heart rate variability in athletes. Sports Medicine, 33(12), 889–919. 10.2165/00007256-200333120-00003 12974657

[phy270917-bib-0006] Bartness, T. J. , Shrestha, Y. B. , Vaughan, C. H. , Schwartz, G. J. , & Song, C. K. (2009). Sensory and sympathetic nervous system control of white adipose tissue lipolysis. Molecular and Cellular Endocrinology, 318(1–2), 34. 10.1016/j.mce.2009.08.031 19747957 PMC2826518

[phy270917-bib-0007] Bellman, J. , Sjöros, T. , Hägg, D. , Atencio Herre, E. , Hieta, J. , Eskola, O. , Laitinen, K. , Nuutila, P. , Jansson, J. O. , Jansson, P. A. , Kalliokoski, K. , Roivainen, A. , & Ohlsson, C. (2024). Loading enhances glucose uptake in muscles, bones, and bone marrow of lower extremities in humans. The Journal of Clinical Endocrinology and Metabolism, 109(12), 3126–3136. 10.1210/clinem/dgae344 38753869 PMC11570666

[phy270917-bib-0008] Bellman, J. , Westerterp, K. , Wouters, L. , Johannesson, M. , Lundqvist, N. , Kullberg, J. , Larsson, C. , Gustafsson, M. , Pettersson, S. , Fridolfsson, J. , Arvidsson, D. , Börjesson, M. , Curiac, D. , Jansson, J. O. , Jansson, P. A. , & Ohlsson, C. (2025). Increased weight‐load improves body composition by reducing fat mass and waist circumference, and by increasing lean mass in participants with obesity: A single‐centre randomised controlled trial. BMC Medicine, 23(1), 317. 10.1186/s12916-025-04143-6 40442671 PMC12123769

[phy270917-bib-0009] Bergfeldt, L. , & Vahedi, F. (2026). Sinoatrial node sympathovagal balance and intrinsic heart rate at rest: no difference between young healthy women and men. American Journal of Physiology. Heart and Circulatory Physiology, 330(4), H955–H960. 10.1152/ajpheart.00001.2026 41651447

[phy270917-bib-0010] Berntson, G. G. , Bigger, T. , Eckberg, D. L. , Grossman, P. , Kaufmann, P. G. , Malik, M. , Nagaraja, H. N. , Porges, S. W. , Saul, J. P. , Stone, P. H. , & Van Der Molen, M. W. (1997). Heart rate variability: Origins, methods, and interpretive caveats: Psychophysiology. Psychophysiology, 34(6), 623–648. 10.1111/j.1469-8986.1997.tb02140.x 9401419

[phy270917-bib-0011] Betts, J. A. , Smith, H. A. , Johnson‐Bonson, D. A. , Ellis, T. I. , Dagnall, J. , Hengist, A. , Carroll, H. , Thompson, D. , Gonzalez, J. T. , & Afman, G. H. (2019). The energy cost of sitting versus standing naturally in man. Medicine and Science in Sports and Exercise, 51(4), 726. 10.1249/MSS.0000000000001841 30673688

[phy270917-bib-0012] Billman, G. E. (2013). The LF/HF ratio does not accurately measure cardiac sympatho‐vagal balance. Frontiers in Physiology, 4, 26. 10.3389/fphys.2013.00026 23431279 PMC3576706

[phy270917-bib-0013] Bootsma, M. , Swenne, C. A. , Janssen, M. J. A. , Cats, V. M. , & Schalij, M. J. (2003). Heart rate variability and sympathovagal balance: Pharmacological validation. Netherlands Heart Journal, 11(6), 250–259. 25696224 PMC2499895

[phy270917-bib-0014] Brockmann, L. , & Hunt, K. J. (2023). Heart rate variability changes with respect to time and exercise intensity during heart‐rate‐controlled steady‐state treadmill running. Scientific Reports, 13(1), 8515. 10.1038/s41598-023-35717-0 37231117 PMC10213066

[phy270917-bib-0015] Carter, J. B. , Banister, E. W. , & Blaber, A. P. (2003). Effect of endurance exercise on autonomic control of heart rate. Sports Medicine, 33(1), 33–46. 10.2165/00007256-200333010-00003 12477376

[phy270917-bib-0016] Cohen, P. , & Kajimura, S. (2021). The cellular and functional complexity of thermogenic fat. Nature Reviews. Molecular Cell Biology, 22(6), 393–409. 10.1038/s41580-021-00350-0 33758402 PMC8159882

[phy270917-bib-0017] Eckberg, D. L. (1997). Sympathovagal balance. Circulation, 96(9), 3224–3232. 10.1161/01.CIR.96.9.3224 9386196

[phy270917-bib-0018] Fisher, J. P. , Young, C. N. , & Fadel, P. J. (2015). Autonomic adjustments to exercise in humans. Comprehensive Physiology, 5(2), 475–512. 10.1002/cphy.c140022 25880502

[phy270917-bib-0019] Fox, S. M. , & Naughton, J. P. (1972). Physical activity and the prevention of coronary heart disease. Preventive Medicine, 1(1), 92–120. 10.1016/0091-7435(72)90079-5 5069016

[phy270917-bib-0020] Franz, R. , Maturana, M. A. , Magalhães, J. A. , Moraes, R. S. , & Spritzer, P. M. (2013). Central adiposity and decreased heart rate variability in postmenopause: A cross‐sectional study. Climacteric, 16(5), 576–583. 10.3109/13697137.2012.745123 23234242

[phy270917-bib-0021] Garthwaite, T. , Sjoros, T. , Koivumaki, M. , Laine, S. , Vaha‐Ypya, H. , Saarenhovi, M. , Kallio, P. , Löyttyniemi, E. , Sievänen, H. , Houttu, N. , Laitinen, K. , Kalliokoski, K. , Vasankari, T. , Knuuti, J. , & Heinonen, I. (2021). Standing is associated with insulin sensitivity in adults with metabolic syndrome. Journal of Science and Medicine in Sport, 24(12), 1255–1260. 10.1016/j.jsams.2021.08.009 34489177

[phy270917-bib-0022] Garthwaite, T. , Sjöros, T. , Laine, S. , Vähä‐Ypyä, H. , Löyttyniemi, E. , Sievänen, H. , Houttu, N. , Laitinen, K. , Kalliokoski, K. , Vasankari, T. , Knuuti, J. , & Heinonen, I. (2022). Effects of reduced sedentary time on cardiometabolic health in adults with metabolic syndrome: A three‐month randomized controlled trial. Journal of Science and Medicine in Sport, 25(7), 579–585. 10.1016/j.jsams.2022.04.002 35487860

[phy270917-bib-0023] Grassi, G. , Biffi, A. , Seravalle, G. , Trevano, F. Q. , Dell'Oro, R. , Corrao, G. , & Mancia, G. (2019). Sympathetic neural overdrive in the obese and overweight state. Hypertens Dallas Tex 1979, 74(2), 349–358. 10.1161/HYPERTENSIONAHA.119.12885 31203727

[phy270917-bib-0024] Guarino, D. , Nannipieri, M. , Iervasi, G. , Taddei, S. , & Bruno, R. M. (2017). The role of the autonomic nervous system in the pathophysiology of obesity. Frontiers in Physiology, 8, 665. 10.3389/fphys.2017.00665 28966594 PMC5606212

[phy270917-bib-0025] Hadase, M. , Azuma, A. , Zen, K. , Asada, S. , Kawasaki, T. , Kamitani, T. , Kawasaki, S. , Sugihara, H. , & Matsubara, H. (2004). Very low frequency power of heart rate variability is a powerful predictor of clinical prognosis in patients with congestive heart failure. Circulation Journal, 68(4), 343–347. 10.1253/circj.68.343 15056832

[phy270917-bib-0026] Hall, J. E. , & Hall, M. E. (2020). Guyton and Hall textbook of medical physiology (14th ed., p. 1152). Elsevier.

[phy270917-bib-0027] Hautala, A. J. , Mäkikallio, T. H. , Kiviniemi, A. , Laukkanen, R. T. , Nissilä, S. , Huikuri, H. V. , & Tulppo, M. P. (2003). Cardiovascular autonomic function correlates with the response to aerobic training in healthy sedentary subjects. American Journal of Physiology. Heart and Circulatory Physiology, 285(4), H1747–H1752. 10.1152/ajpheart.00202.2003 12816748

[phy270917-bib-0028] Heffernan, K. S. , Fahs, C. A. , Shinsako, K. K. , Jae, S. Y. , & Fernhall, B. (2007). Heart rate recovery and heart rate complexity following resistance exercise training and detraining in young men. American Journal of Physiology. Heart and Circulatory Physiology, 293(5), H3180–H3186. 10.1152/ajpheart.00648.2007 17890428

[phy270917-bib-0029] Jansson, J. O. , Palsdottir, V. , Hagg, D. A. , Schele, E. , Dickson, S. L. , Anesten, F. , Bake, T. , Montelius, M. , Bellman, J. , Johansson, M. E. , & Cone, R. D. (2018). Body weight homeostat that regulates fat mass independently of leptin in rats and mice. Proceedings of the National Academy of Sciences, 115(2), 427–432. 10.1073/pnas.1715687114 PMC5777058 29279372

[phy270917-bib-0030] Kaikkonen, K. M. , Korpelainen, R. I. , Tulppo, M. P. , Kaikkonen, H. S. , Vanhala, M. L. , Kallio, M. A. , Keinänen‐Kiukaanniemi, S. M. , & Korpelainen, J. T. (2014). Physical activity and aerobic fitness are positively associated with heart rate variability in obese adults. Journal of Physical Activity & Health, 11(8), 1614–1621. 10.1123/jpah.2012-0405 24508687

[phy270917-bib-0031] Karvonen, M. J. , Kentala, E. , & Mustala, O. (1957). The effects of training on heart rate: A longitudinal study. Annales Medicinae Experimentalis et Biologiae Fenniae, 35, 307–315. 13470504

[phy270917-bib-0032] Kingsley, J. D. , & Figueroa, A. (2016). Acute and training effects of resistance exercise on heart rate variability. Clinical Physiology and Functional Imaging, 36(3), 179–187. 10.1111/cpf.12223 25524332

[phy270917-bib-0033] Kiviniemi, A. M. , Perkiömäki, N. , Auvinen, J. , Niemelä, M. , Tammelin, T. , Puukka, K. , Ruokonen, A. , Keinänen‐Kiukaanniemi, S. , Tulppo, M. P. , Järvelin, M. R. , Jämsä, T. , Huikuri, H. V. , & Korpelainen, R. (2017). Fitness, fatness, physical activity, and autonomic function in midlife. Medicine and Science in Sports and Exercise, 49(12), 2459–2468. 10.1249/MSS.0000000000001387 29135784

[phy270917-bib-0034] Koenig, J. , & Thayer, J. F. (2016). Sex differences in healthy human heart rate variability: A meta‐analysis. Neuroscience and Biobehavioral Reviews, 64, 288–310. 10.1016/j.neubiorev.2016.03.007 26964804

[phy270917-bib-0035] Lambert, G. W. , Schlaich, M. P. , Eikelis, N. , & Lambert, E. A. (2019). Sympathetic activity in obesity: A brief review of methods and supportive data. Annals of the New York Academy of Sciences, 1454(1), 56–67. 10.1111/nyas.14140 31268175

[phy270917-bib-0036] Li, X. , Shaffer, M. L. , Rodriguez‐Colon, S. , He, F. , Wolbrette, D. L. , Alagona, P., Jr. , Wu, C. , & Liao, D. (2011). The circadian pattern of cardiac autonomic modulation in a middle‐aged population. Clinical Autonomic Research, 21(3), 143. 10.1007/s10286-010-0112-4 21240538 PMC3093547

[phy270917-bib-0037] Liu, B. , Du, Y. , Wu, Y. , Snetselaar, L. G. , Wallace, R. B. , & Bao, W. (2021). Trends in obesity and adiposity measures by race or ethnicity among adults in the United States 2011‐18: Population based study. The BMJ, 372, n365. 10.1136/bmj.n365 33727242 PMC7961695

[phy270917-bib-0038] Magkos, F. , Sørensen, T. I. A. , Raubenheimer, D. , Dhurandhar, N. V. , Loos, R. J. F. , & Bosy‐Westphal, A. (2024). On the pathogenesis of obesity: Causal models and missing pieces of the puzzle. Nature Metabolism, 6(10), 1856–1865. 10.1038/s42255-024-01106-8 39164418

[phy270917-bib-0039] Malik, M. , Bigger, J. T. , Camm, A. J. , Kleiger, R. E. , Malliani, A. , Moss, A. J. , & Schwartz, P. J. (1996). Heart rate variability ‐ standards of measurement, physiological interpretation, and clinical use. European Heart Journal, 17, 354–381. 8737210

[phy270917-bib-0040] Michael, S. , Graham, K. S. , & Davis, G. M. (2017). Cardiac autonomic responses during exercise and post‐exercise recovery using heart rate variability and systolic time intervals—A review. Frontiers in Physiology, 8, 301. 10.3389/fphys.2017.00301 28611675 PMC5447093

[phy270917-bib-0041] Ohlsson, C. , Gidestrand, E. , Bellman, J. , Larsson, C. , Palsdottir, V. , Hagg, D. , Jansson, P. A. , & Jansson, J. O. (2020). Increased weight loading reduces body weight and body fat in obese subjects—A proof of concept randomized clinical trial. EClinicalMedicine, 22, 100338. 10.1016/j.eclinm.2020.100338 32510046 PMC7264953

[phy270917-bib-0042] Oliveira, C. , Silveira, E. A. , Rosa, L. , Santos, A. , Rodrigues, A. P. , Mendonça, C. , Silva, L. , Gentil, P. , & Rebelo, A. C. (2020). Risk factors associated with cardiac autonomic modulation in obese individuals. Journal of Obesity, 2020, 7185249. 10.1155/2020/7185249 32318288 PMC7152942

[phy270917-bib-0043] Phelps, N. H. , Singleton, R. K. , Zhou, B. , Heap, R. A. , Mishra, A. , Bennett, J. E. , Paciorek, C. J. , Lhoste, V. P. F. , Carrillo‐Larco, R. M. , Stevens, G. A. , Rodriguez‐Martinez, A. , Bixby, H. , Bentham, J. , di Cesare, M. , Danaei, G. , Rayner, A. W. , Barradas‐Pires, A. , Cowan, M. J. , Savin, S. , … Riley, L. M. (2024). Worldwide trends in underweight and obesity from 1990 to 2022: A pooled analysis of 3663 population‐representative studies with 222 million children, adolescents, and adults. The Lancet, 403, 1027–1050. 10.1016/S0140-6736(23)02750-2 PMC7615769 38432237

[phy270917-bib-0044] Qiu, Y. , Fernández‐García, B. , Lehmann, H. I. , Li, G. , Kroemer, G. , López‐Otín, C. , & Xiao, J. (2023). Exercise sustains the hallmarks of health. Journal of Sport and Health Science, 12(1), 8–35. 10.1016/j.jshs.2022.10.003 36374766 PMC9923435

[phy270917-bib-0045] Russo, B. , Menduni, M. , Borboni, P. , Picconi, F. , & Frontoni, S. (2021). Autonomic nervous system in obesity and insulin‐resistance—The complex interplay between leptin and central nervous system. International Journal of Molecular Sciences, 22(10), 5187. 10.3390/ijms22105187 34068919 PMC8156658

[phy270917-bib-0046] Salih, T. R. , & Abdulateef, D. S. (2026). Correlation between heart rate variability and estradiol, progesterone, and the estradiol/progesterone ratio across menstrual phases in healthy women. Physiological Reports, 14(8), e70887. 10.14814/phy2.70887 42033060 PMC13109644

[phy270917-bib-0047] Sammito, S. , Sammito, W. , & Böckelmann, I. (2016). The circadian rhythm of heart rate variability. Biological Rhythm Research, 47(5), 717–730. 10.1080/09291016.2016.1183887

[phy270917-bib-0048] Saxton, S. N. , Withers, S. B. , & Heagerty, A. M. (2019). Emerging roles of sympathetic nerves and inflammation in perivascular adipose tissue. Cardiovascular Drugs and Therapy, 33(2), 245–259. 10.1007/s10557-019-06862-4 30747398 PMC6509065

[phy270917-bib-0049] Schmalenberger, K. M. , Eisenlohr‐Moul, T. A. , Jarczok, M. N. , Eckstein, M. , Schneider, E. , Brenner, I. G. , Duffy, K. , Schweizer, S. , Kiesner, J. , Thayer, J. F. , & Ditzen, B. (2020). Menstrual cycle changes in vagally‐mediated heart rate variability are associated with progesterone: Evidence from two within‐person studies. Journal of Clinical Medicine, 9(3), 617. 10.3390/jcm9030617 32106458 PMC7141121

[phy270917-bib-0050] Schmalenberger, K. M. , Eisenlohr‐Moul, T. A. , Würth, L. , Schneider, E. , Thayer, J. F. , Ditzen, B. , & Jarczok, M. N. (2019). A systematic review and meta‐analysis of within‐person changes in cardiac vagal activity across the menstrual cycle: Implications for female health and future studies. Journal of Clinical Medicine, 8(11), 1946. 10.3390/jcm8111946 31726666 PMC6912442

[phy270917-bib-0051] Schulze, M. B. , & Stefan, N. (2024). Metabolically healthy obesity: From epidemiology and mechanisms to clinical implications. Nature Reviews Endocrinology, 20, 1–14. 10.1038/s41574-024-01008-5 38937638

[phy270917-bib-0052] Shaffer, F. , & Ginsberg, J. P. (2017). An overview of heart rate variability metrics and norms. Frontiers in Public Health, 5, 258. 10.3389/fpubh.2017.00258 29034226 PMC5624990

[phy270917-bib-0053] Shuval, K. , Barlow, C. E. , Finley, C. E. , Gabriel, K. P. , Schmidt, M. D. , & DeFina, L. F. (2015). Standing, obesity, and metabolic syndrome: Findings from the Cooper Center longitudinal study. Mayo Clinic Proceedings, 90(11), 1524–1532. 10.1016/j.mayocp.2015.07.022 26422243

[phy270917-bib-0054] Smith, M. M. , & Minson, C. T. (2012). Obesity and adipokines: Effects on sympathetic overactivity. The Journal of Physiology, 590(8), 1787. 10.1113/jphysiol.2011.221036 22351630 PMC3573303

[phy270917-bib-0055] Souza, H. C. D. , Philbois, S. V. , Veiga, A. C. , & Aguilar, B. A. (2021). Heart rate variability and cardiovascular fitness: What we know so far. Vascular Health and Risk Management, 17, 701–711. 10.2147/VHRM.S279322 34803382 PMC8598208

[phy270917-bib-0056] Speakman, J. R. , & Elmquist, J. K. (2022). Obesity: An evolutionary context. Life Metabolism, 1(1), 10–24. 10.1093/lifemeta/loac002 36394061 PMC9642988

[phy270917-bib-0057] Strüven, A. , Holzapfel, C. , Stremmel, C. , & Brunner, S. (2021). Obesity, nutrition and heart rate variability. International Journal of Molecular Sciences, 22(8), 4215. 10.3390/ijms22084215 33921697 PMC8072942

[phy270917-bib-0058] Thayer, J. F. , & Lane, R. D. (2009). Claude Bernard and the heart‐brain connection: Further elaboration of a model of neurovisceral integration. Neuroscience and Biobehavioral Reviews, 33(2), 81–88. 10.1016/j.neubiorev.2008.08.004 18771686

[phy270917-bib-0059] Thorp, A. A. , & Schlaich, M. P. (2015). Relevance of sympathetic nervous system activation in obesity and metabolic syndrome. Journal of Diabetes Research, 2015(1), 341583. 10.1155/2015/341583 26064978 PMC4430650

[phy270917-bib-0060] Tiwari, R. , Kumar, R. , Malik, S. , Raj, T. , & Kumar, P. (2021). Analysis of heart rate variability and implication of different factors on heart rate variability. Current Cardiology Reviews, 17(5), e160721189770. 10.2174/1573403X16999201231203854 33390146 PMC8950456

[phy270917-bib-0061] Wang, Y. , Leung, V. H. , Zhang, Y. , Nudell, V. S. , Loud, M. , Servin‐Vences, M. R. , Yang, D. , Wang, K. , Moya‐Garzon, M. D. , Li, V. L. , Long, J. Z. , Patapoutian, A. , & Ye, L. (2022). The role of somatosensory innervation of adipose tissues. Nature, 609(7927), 569–574. 10.1038/s41586-022-05137-7 36045288 PMC9477745

[phy270917-bib-0062] World Health Organization . (2024). Obesity and overweight [Internet]. https://www.who.int/news‐room/fact‐sheets/detail/obesity‐and‐overweight

