# Supplementary material for: Short‐term effects of weight‐loading on heart rate variability in obese adults
Source: Physiol Rep. 2026 May 21;14(10):e70917. doi: 10.14814/phy2.70917 (PMC13239273; doi:10.14814/phy2.70917)
Supplement: Supplementary file 1 — Figure S1: Baseline time domain values. Figure S2: Baseline frequency domain values. Table S1: Baseline characteristics for additional parameters not included in main manuscript. Table S2: Absolute changes from baseline. Table S3: Relative changes from baseline. Table S4: Sex‐stratified analyses: Females absolute changes. Table S5: Sex‐stratified analyses: Males absolute changes. [file PHY2-14-e70917-s002.docx]

Supplementary Information

Short-term effects of weight-loading on heart rate variability in obese adults

**Authors**

Jakob Bellman, Per-Anders Jansson, John-Olov Jansson, Claes Ohlsson, Lennart Bergfeldt

**Contents**

| *Supplementary figures* | |
| --- | --- |
|  | |
| **Figure S1** | Baseline time domain values |
|  |  |
| **Figure S2** | Baseline frequency domain values |
|  |  |
|  |  |
| *Supplementary tables* | |
|  | |
| **Table S1** | Baseline characteristics for additional parameters not included in main manuscript |
|  |  |
| **Table S2** | Absolute changes from baseline |
|  |  |
| **Table S3** | Relative changes from baseline |
|  |  |
| **Table S4** | Sex-stratified analyses: females absolute changes |
|  |  |
| **Table S5** | Sex-stratified analyses: males absolute changes |

# Supplementary Figures


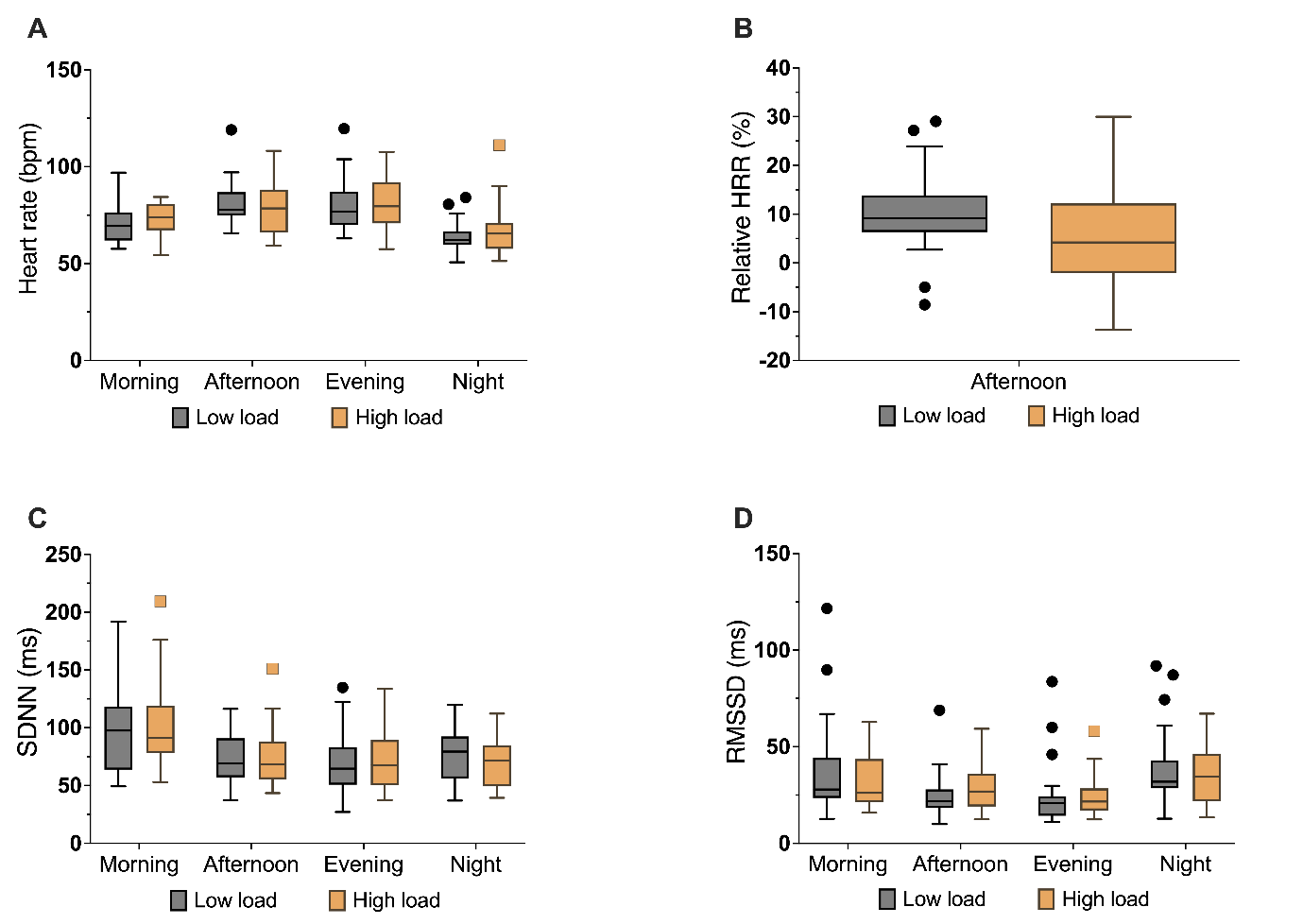


**Figure S1. Baseline (Day −13) time domain HRV parameters showing circadian variation prior to vest use** in the low-load (n = 26) and high-load (n = 20–23) groups. Panels show **(A)** heart rate, **(B)** relative HRR (%HRR), **(C)** SDNN, and **(D)** RMSSD. No significant between-group differences were observed. Box plots show medians (lines), interquartile ranges (boxes), whiskers (Tukey’s method), and outliers (points).

**Abbreviations:** are provided as shown in Table 1.

**
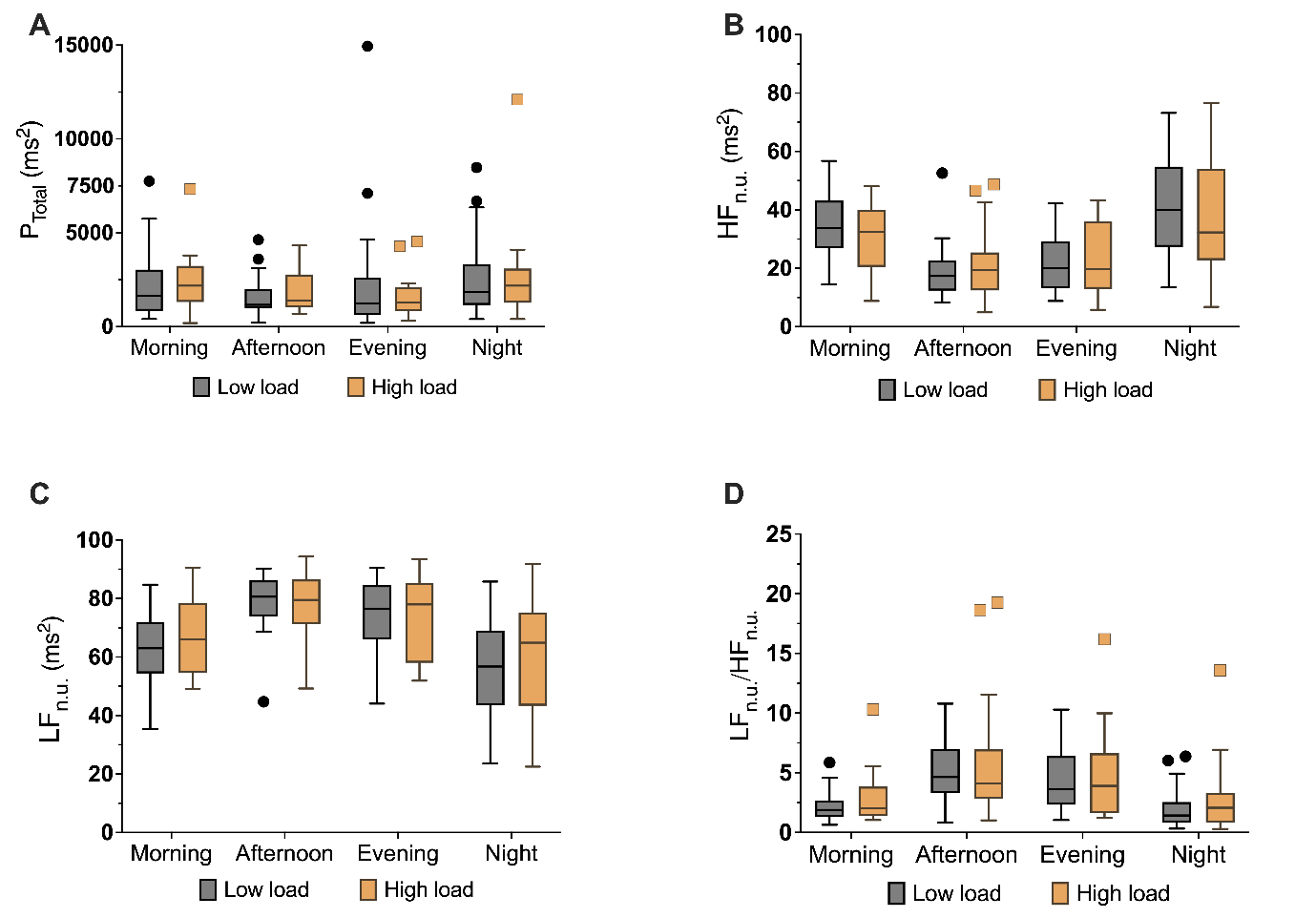
**

**Figure S2. Baseline (Day −13) frequency domain HRV parameters showing circadian variation prior to vest use** in the low-load (n = 25) and high-load (n = 19–24) groups. Panels show **(A)** total power spectral density (P_Total_), **(B)** normalized high-frequency power (HF_n.u._), **(C)** normalized low-frequency power (LF_n.u._), and **(D)** LF_n.u._/HF_n.u._ ratio. No baseline group differences were observed. Box plots show medians (lines), interquartile ranges (boxes), whiskers (Tukey’s method), and outliers (points).

**Abbreviations:** are provided as shown in Table 1.

# Supplementary Tables

## Table S1 – Baseline characteristics for additional parameters

| **Characteristics** | **Low Load** | | | **High Load** | | | **P-value (Mann-Whitney U)** |
| --- | --- | --- | --- | --- | --- | --- | --- |
|  | **Median** | **IQR** | **n** | **Median** | **IQR** | **n** |  |
| **Time Domain (ms)** |  |  |  |  |  |  |  |
| SDANN, 24h | 129 | 41 | 26 | 119 | 43 | 23 | NS |
| SDANN, Morning | 63 | 44 | 26 | 72 | 46 | 23 | NS |
| SDANN, Afternoon | 39 | 28 | 26 | 41 | 29 | 23 | NS |
| SDANN, Evening | 42 | 27 | 26 | 41 | 42 | 20 | NS |
| SDANN, Night | 35 | 15 | 26 | 27 | 26 | 20 | NS |
|  |  |  |  |  |  |  |  |
| Triangular Index. 24h | 11 | 3 | 26 | 11 | 4 | 23 | NS |
|  |  |  |  |  |  |  |  |
| **Frequency Domain (ms^2^)** |  |  |  |  |  |  |  |
| P_HF_, morning | 336 | 793 | 25 | 375 | 543 | 24 | NS |
| P_HF_, afternoon | 142 | 155 | 25 | 229 | 235 | 20 | NS |
| P_HF_, evening | 154 | 286 | 25 | 150 | 176 | 21 | NS |
| P_HF_, night | 458 | 576 | 25 | 465 | 799 | 20 | NS |
|  |  |  |  |  |  |  |  |
| P_LF,_ morning | 742 | 1124 | 25 | 1051 | 975 | 24 | NS |
| P_LF_, afternoon | 712 | 595 | 25 | 940 | 913 | 20 | NS |
| P_LF_, evening | 657 | 1191 | 25 | 717 | 879 | 21 | NS |
| P_LF_, night | 658 | 1067 | 25 | 913 | 689 | 20 | NS |

**Table S1. Baseline characteristics for additional HRV parameters.** Values are medians with interquartile ranges (IQR) for all participants who completed the trial according to protocol. Time domain measures were analyzed over the full 24-hour ECG recording, and both time- and frequency domain measures during four predefined 1-hour periods (morning, afternoon, evening, night).

**Abbreviations:** are provided as shown in Table 1.

## Table S2 – Absolute changes from baseline

|  | **Low Load** | | | **High Load** | | | **P-value (Mann-Whitney U)** |
| --- | --- | --- | --- | --- | --- | --- | --- |
|  | **Median** | **IQR** | **n** | **Median** | **IQR** | **n** |  |
| HR, 24h (bpm) | 0.4 | 5.4 | 26 | 3.3 | 7.9 | 23 | 0.406 |
| HR, Morning (bpm) | 0.4 | 7.9 | 26 | 3.2 | 7.3 | 23 | 0.401 |
| HR, Afternoon (bpm) | 0.2 | 14.5 | 26 | 4.9 | 10.5 | 23 | 0.060 |
| HR, Evening (bpm) | 0.8 | 11.9 | 26 | 3.6 | 12.2 | 20 | 0.376 |
| HR, Night (bpm) | 1.5 | 6.1 | 26 | 1.2 | 8.5 | 20 | 0.969 |
|  |  |  |  |  |  |  |  |
| HRR (bpm) | -0.4 | 7.9 | 26 | -3.2 | 7.3 | 23 | 0.400 |
| Relative HRR (%HRR) | -1.5% | 14.8% | 26 | **4.8% **** | 10.6% | 23 | **0.032** |
|  |  |  |  |  |  |  |  |
| Systolic Blood Pressure (mmHg) | -4.0 | 11.5 | 26 | -3.0 | 9.0 | 25 | 0.754 |
| Diastolic Blood Pressure (mmHg) | 0.5 | 11.3 | 26 | 1.0 | 8.0 | 25 | 0.220 |
|  |  |  |  |  |  |  |  |
| **Time Domain (ms)** |  |  |  |  |  |  |  |
| SDNN, 24h | -0.4 | 31 | 26 | 10 | 40 | 23 | 0.142 |
| SDNN, Morning | **25 *** | 29 | 26 | 12 | 39 | 23 | 0.100 |
| SDNN, Afternoon | **8 *** | 24 | 26 | -7 | 35 | 23 | **0.010** |
| SDNN, Evening | -1 | 21 | 26 | -5 | 30 | 20 | 0.273 |
| SDNN, Night | -5 | 31 | 26 | -6 | 31 | 20 | 0.763 |
|  |  |  |  |  |  |  |  |
| RMSSD 24h | -1 | 6 | 26 | 0.3 | 10 | 23 | 0.847 |
| RMSSD, Morning | 0.3 | 11 | 26 | -1 | 9 | 23 | >0.999 |
| RMSSD, Afternoon | 1 | 8 | 26 | **-3 *** | 14 | 23 | **0.002** |
| RMSSD, Evening | 0.1 | 8 | 26 | **-3 *** | 12 | 20 | **0.013** |
| RMSSD, Night | -1 | 15 | 26 | -2 | 16 | 20 | 0.641 |
|  |  |  |  |  |  |  |  |
| SDANN, 24h | 2 | 32 | 26 | 12 | 42 | 23 | 0.193 |
| SDANN, Morning | **17 *** | 46 | 26 | 1 | 53 | 23 | 0.190 |
| SDANN, Afternoon | 7 | 44 | 26 | 1 | 37 | 23 | 0.161 |
| SDANN, Evening | -0.5 | 31 | 26 | 0.4 | 30 | 20 | 0.564 |
| SDANN, Night | 1 | 24 | 26 | -3 | 26 | 20 | 0.780 |
|  |  |  |  |  |  |  |  |
| Triangular Index, 24h | -0.4 | 2.6 | 26 | 2 | 6.0 | 23 | 0.152 |
|  |  |  |  |  |  |  |  |
| **Frequency Domain (ms^2^)** |  |  |  |  |  |  |  |
| P_Total,_ Morning | **369 *** | 1267 | 25 | -30 | 1649 | 24 | 0.270 |
| P_Total,_ Afternoon | 46 | 894 | 25 | -241 | 1096 | 20 | **0.022** |
| P_Total,_ evening | 79 | 848 | 25 | -10 | 1183 | 21 | 0.678 |
| P_Total,_ night | -77 | 1920 | 25 | 218 | 1900 | 20 | 0.760 |
|  |  |  |  |  |  |  |  |
| P_HF,_ morning | 22 | 205 | 25 | 28 | 299 | 24 | 0.699 |
| P_HF,_ afternoon | 18 | 214 | 25 | **-61 *** | 190 | 20 | 0.009 |
| P_HF,_ evening | 8 | 177 | 25 | -32 | 162 | 21 | 0.219 |
| P_HF,_ night | -39 | 573 | 25 | -52 | 426 | 20 | 0.519 |
|  |  |  |  |  |  |  |  |
| P_LF,_ morning | 172 | 672 | 25 | -15 | 813 | 24 | 0.316 |
| P_LF,_ afternoon | -16 | 462 | 25 | -198 | 750 | 20 | **0.036** |
| P_LF,_ evening | 14 | 443 | 25 | -46 | 773 | 21 | 0.743 |
| P_LF,_ night | 15 | 471 | 25 | -8 | 714 | 20 | 0.830 |
|  |  |  |  |  |  |  |  |
| **Frequency Domain** |  |  |  |  |  |  |  |
| **Normalized values (n.u.; ms^2^)** |  |  |  |  |  |  |  |
| P_HF_ n.u., morning | -3 | 13 | 25 | -2 | 13 | 24 | 0.388 |
| P_HF_ n.u., afternoon | 1 | 10 | 25 | -3 | 8 | 20 | 0.130 |
| P_HF_ n.u., evening | 2 | 7 | 25 | -0.5 | 18 | 21 | 0.211 |
| P_HF_ n.u., night | 3 | 15 | 25 | -8 | 16 | 20 | 0.081 |
|  |  |  |  |  |  |  |  |
| P_LF_ n.u., morning | **6 *** | 12 | 25 | 2 | 12 | 24 | 0.244 |
| P_LF_ n.u., afternoon | -1 | 12 | 25 | 2 | 11 | 20 | 0.226 |
| P_LF_ n.u., evening | -1 | 9 | 25 | 2 | 24 | 21 | 0.346 |
| P_LF_ n.u., night | -4 | 17 | 25 | 8 | 16 | 20 | 0.108 |
|  |  |  |  |  |  |  |  |
| Ratio P_LF_/P_HF_ n.u., morning | 0.4 | 1.3 | 25 | 0.2 | 1.6 | 24 | 0.345 |
| Ratio P_LF_/P_HF_ n.u., afternoon | -0.2 | 2.9 | 25 | 1.2 | 2.5 | 20 | 0.069 |
| Ratio P_LF_/P_HF_ n.u., evening | -0.2 | 1.9 | 25 | 0.5 | 4.1 | 21 | 0.160 |
| Ratio P_LF_/P_HF_ n.u., night | -0.1 | 1.4 | 25 | 0.5 | 1.9 | 20 | 0.191 |

**Table S2. Absolute change from baseline in cardiovascular and HRV parameters.** Values are medians with interquartile ranges (IQR) for all participants who completed the protocol. HRV parameters were derived from 24-hour ECG recordings, and blood pressure measured in the morning. Time- and frequency domain measures were analyzed over predefined 1-hour periods (morning, afternoon, evening, night). Statistically significant within-group changes are indicated in bold. **P < 0.05, **P < 0.01.*

**Abbreviations:** are provided as shown in Table 1.

## Table S3 – Relative changes from baseline

|  | **Low Load** | | | **High Load** | | | **P-value (Mann-Whitney U)** |
| --- | --- | --- | --- | --- | --- | --- | --- |
|  | **Median** | **IQR** | **n** | **Median** | **IQR** | **n** |  |
| **Time Domain (%)** |  |  |  |  |  |  |  |
| HR, 24h | 0.5% | 7.0% | 26 | 4.1% | 10.6% | 23 | 0.355 |
| HR, Morning | 0.4% | 12.6% | 26 | 4.6% | 11.2% | 23 | 0.421 |
| HR, Afternoon | 0.1% | 17.7% | 26 | **6.9% **** | 14.9% | 23 | **0.0496** |
| HR, Evening | 1.1% | 14.3% | 26 | 5.0% | 14.5% | 20 | 0.339 |
| HR, Night | 2.3% | 10.3% | 26 | 2.0% | 13.3% | 20 | 0.939 |
| HRR | -0.3% | 7.0% | 26 | -3.0% | 7.7% | 24 | 0.250 |
| Relative HRR (%HRR) | -14.7% | 133.2% | 26 | 39.0% | 194.8% | 23 | 0.278 |
| Systolic Blood Pressure | -3.1% | 9.5% | 26 | -2.6% | 7.1% | 25 | 0.688 |
| Diastolic Blood Pressure | 0.6% | 15.3% | 26 | 1.2% | 12.5% | 25 | 0.228 |
|  |  |  |  |  |  |  |  |
| SDNN, 24h | -0.6% | 21.3% | 26 | 7.0% | 31.1% | 23 | 0.148 |
| SDNN, Morning | **30.6% **** | 33.0% | 26 | 13.9% | 40.1% | 23 | 0.063 |
| SDNN, Afternoon | **12.3% *** | 43.6% | 26 | -7.4% | 45.9% | 23 | **0.020** |
| SDNN, Evening | 0.1% | 32.8% | 26 | -5.6% | 46.1% | 20 | 0.373 |
| SDNN, Night | -7.3% | 38.0% | 26 | -7.9% | 46.5% | 20 | 0.904 |
|  |  |  |  |  |  |  |  |
| RMSSD, 24h | -2.8% | 17.5% | 26 | 1.3% | 25.7% | 23 | 0.893 |
| RMSSD, Morning | 0.6% | 43.7% | 26 | -3.1% | 35.3% | 23 | 0.773 |
| RMSSD, Afternoon | 6.8% | 32.9% | 26 | **-17.2% **** | 47.7% | 23 | **0.003** |
| RMSSD, Evening | 0.2% | 36.3% | 26 | -18.6% | 40.2% | 20 | **0.006** |
| RMSSD, Night | -2.8% | 47.1% | 26 | -6.3% | 48.4% | 20 | 0.576 |
|  |  |  |  |  |  |  |  |
| SDANN, 24h | 1.8% | 25.1% | 26 | 8.3% | 39.5% | 23 | 0.148 |
| SDANN, Morning | **32.3% **** | 102.8% | 26 | 1.1% | 64.9% | 23 | 0.131 |
| SDANN, Afternoon | 22.3% | 132.6% | 26 | 4.5% | 87.6% | 23 | 0.228 |
| SDANN, Evening | -4.0% | 92.6% | 26 | 1.0% | 71.7% | 20 | 0.717 |
| SDANN, Night | 2.8% | 69.2% | 26 | -9.9% | 124.1% | 20 | 0.869 |
|  |  |  |  |  |  |  |  |
| Triangular Index, 24h | -3.6% | 22.7% | 26 | 17.5% | 43.4% | 23 | 0.112 |
|  |  |  |  |  |  |  |  |
| **Frequency Domain (%)** |  |  |  |  |  |  |  |
| P_Total,_ Morning | 36.8% | 91.0% | 25 | -3.0% | 82.6% | 24 | 0.099 |
| P_Total,_ Afternoon | 2.7% | 81.9% | 25 | -17.0% | 57.7% | 20 | 0.050 |
| P_Total,_ evening | 6.4% | 96.5% | 25 | -0.5% | 90.3% | 21 | 0.358 |
| P_Total,_ night | -0.9% | 89.1% | 25 | 17.2% | 98.9% | 20 | 0.991 |
|  |  |  |  |  |  |  |  |
| P_HF,_ morning | 13.0% | 98.3% | 25 | 9.6% | 99.2% | 24 | 0.744 |
| P_HF,_ afternoon | 9.3% | 137.8% | 25 | **-27.4% *** | 73.0% | 20 | **0.011** |
| P_HF,_ evening | 19.7% | 147.3% | 25 | -26.8% | 68.5% | 21 | 0.085 |
| P_HF,_ night | -9.7% | 147.8% | 25 | -18.7% | 79.1% | 20 | 0.395 |
|  |  |  |  |  |  |  |  |
| P_LF,_ morning | 18.7% | 132.3% | 25 | -0.1% | 88.5% | 24 | 0.199 |
| P_LF,_ afternoon | -2.1% | 58.1% | 25 | -21.4% | 69.0% | 20 | **0.021** |
| P_LF,_ evening | 3.9% | 100.1% | 25 | -10.5% | 92.0% | 21 | 0.406 |
| P_LF,_ night | 2.7% | 73.7% | 25 | -0.3% | 115.7% | 20 | 0.795 |
|  |  |  |  |  |  |  |  |
| **Frequency Domain** |  |  |  |  |  |  |  |
| **Normalized values (%)** |  |  |  |  |  |  |  |
| P_HF_ n.u., morning | -10.2% | 37.8% | 25 | -6.8% | 46.1% | 24 | 0.519 |
| P_HF_ n.u., afternoon | 5.4% | 55.5% | 25 | -18.4% | 41.8% | 20 | 0.073 |
| P_HF_ n.u., evening | 8.0% | 42.8% | 25 | -4.2% | 72.2% | 21 | 0.166 |
| P_HF_ n.u., night | 4.2% | 53.7% | 25 | -20.2% | 47.0% | 20 | 0.103 |
|  |  |  |  |  |  |  |  |
| P_LF_ n.u., morning | **9.4% *** | 20.0% | 25 | 4.0% | 18.1% | 24 | 0.270 |
| P_LF_ n.u., afternoon | -1.2% | 15.4% | 25 | 1.9% | 14.1% | 20 | 0.281 |
| P_LF_ n.u., evening | -2.0% | 12.5% | 25 | 2.2% | 34.3% | 21 | 0.418 |
| P_LF_ n.u., night | -7.1% | 26.1% | 25 | 12.1% | 35.5% | 20 | 0.103 |
|  |  |  |  |  |  |  |  |
| Ratio P_LF_/P_HF_ n.u., morning | **18.1% *** | 77.0% | 25 | 10.9% | 77.3% | 24 | 0.388 |
| Ratio P_LF_/P_HF_ n.u., afternoon | -6.3% | 65.6% | 25 | 26.0% | 157.4% | 20 | 0.066 |
| Ratio P_LF_/P_HF_ n.u., evening | -8.7% | 58.5% | 25 | 6.7% | 151.1% | 21 | 0.211 |
| Ratio P_LF_/P_HF_ n.u., night | -15.1% | 88.2% | 25 | **50.1% *** | 110.3% | 20 | 0.142 |

**Table S3. Relative change from baseline in cardiovascular and HRV parameters.** Values are medians with interquartile ranges (IQR) expressed as percent change for all participants who completed the protocol. HRV parameters were derived from 24-hour ECG recordings, and blood pressure measured in the morning. Time- and frequency domain measures were analyzed over predefined 1-hour periods (morning, afternoon, evening, night). Statistically significant within-group changes are shown in bold. **P < 0.05, **P < 0.01.*

**Abbreviations:** are provided as shown in Table 1.

## Table S4 – Sex-stratified analyses: females absolute changes from baseline

|  | **Low Load** | | | **High Load** | | | **P-value (Mann- Whitney U)** |
| --- | --- | --- | --- | --- | --- | --- | --- |
|  | **Median** | **IQR** | **n** | **Median** | **IQR** | **n** |  |
|  |  |  |  |  |  |  |  |
| HR, 24h | -2 | 6 | 13 | 3 | 9 | 13 | 0.248 |
| HR, Morning | 1 | 13 | 13 | 4 | 8 | 13 | 0.570 |
| HR, Afternoon | 0.3 | 10 | 13 | **6 **** | 8 | 13 | **0.008** |
| HR, Evening | 3 | 9 | 13 | 5 | 10 | 11 | 0.252 |
| HR, Night | 2 | 8 | 13 | **5 *** | 6 | 11 | 0.154 |
| HRR | -1 | 13 | 13 | -4 | 8 | 13 | 0.570 |
| Relative HRR (%HRR) | -3.2% | 13.8% | 13 | **5.8% **** | 7.8% | 13 | **0.012** |
| Systolic Blood Pressure | -4 | 9 | 13 | -2 | 7 | 14 | 0.434 |
| Diastolic Blood Pressure | 2 | 8 | 13 | 3 | 9 | 14 | 0.378 |
|  |  |  |  |  |  |  |  |
| **Time Domain (ms)** |  |  |  |  |  |  |  |
| SDNN, 24h | -2 | 24 | 13 | -1 | 37 | 13 | 0.801 |
| SDNN, Morning | 19 | 43 | 13 | 12 | 47 | 13 | 0.264 |
| SDNN, Afternoon | 6 | 25 | 13 | -7 | 34 | 13 | 0.243 |
| SDNN, Evening | -4 | 15 | 13 | -6 | 30 | 11 | 0.733 |
| SDNN, Night | 5 | 33 | 13 | -7 | 47 | 11 | 0.820 |
|  |  |  |  |  |  |  |  |
| RMSSD, 24h | -1 | 9 | 13 | -1 | 11 | 13 | 0.587 |
| RMSSD, Morning | -2 | 26 | 13 | -3 | 9 | 13 | 0.733 |
| RMSSD, Afternoon | 2 | 10 | 13 | **-8 *** | 12 | 13 | **0.005** |
| RMSSD, Evening | 1 | 7 | 13 | -4 | 11 | 11 | **0.021** |
| RMSSD, Night | -0.2 | 16 | 13 | -8 | 12 | 11 | 0.277 |
|  |  |  |  |  |  |  |  |
| **Frequency Domain** |  |  |  |  |  |  |  |
| **Normalized values (n.u.; ms^2^)** |  |  |  |  |  |  |  |
| P_HF_ n.u., morning | -3 | 12 | 13 | -5 | 9 | 13 | 0.840 |
| P_HF_ n.u., afternoon | -1 | 12 | 13 | **-6 **** | 9 | 10 | **0.021** |
| P_HF_ n.u., evening | 0.3 | 7 | 13 | -5 | 24 | 11 | 0.277 |
| P_HF_ n.u., night | 5 | 26 | 13 | -9 | 22 | 11 | 0.303 |
|  |  |  |  |  |  |  |  |
| P_LF_ n.u., morning | 6 | 10 | 13 | 5 | 10 | 13 | 0.724 |
| P_LF_ n.u., afternoon | 1 | 11 | 13 | **8 *** | 13 | 10 | **0.049** |
| P_LF_ n.u., evening | 0.4 | 13 | 13 | 9 | 30 | 11 | 0.531 |
| P_LF_ n.u., night | -4 | 29 | 13 | 8 | 23 | 11 | 0.331 |
|  |  |  |  |  |  |  |  |
| Ratio P_LF_/P_HF_ n.u., morning | **0.4 *** | 0.9 | 13 | **0.3 *** | 0.9 | 13 | 0.960 |
| Ratio P_LF_/P_HF_ n.u., afternoon | 0.3 | 3.5 | 13 | **2.0 **** | 4.3 | 10 | **0.026** |
| Ratio P_LF_/P_HF_ n.u., evening | -0.1 | 1.9 | 13 | **2.7 *** | 4.9 | 11 | 0.093 |
| Ratio P_LF_/P_HF_ n.u., night | -0.1 | 1.5 | 13 | 0.5 | 2.0 | 11 | 0.186 |

**Table S4. Absolute change from baseline in cardiovascular and HRV parameters among females.** Values are medians with interquartile ranges (IQR) for all females who completed the protocol. HRV parameters were derived from 24-hour ECG recordings, and blood pressure measured in the morning. Time- and frequency domain measures were analyzed over predefined 1-hour periods (morning, afternoon, evening, night). Statistically significant within-group changes are shown in bold. **P < 0.05, **P < 0.01.*

**Abbreviations:** are provided as shown in Table 1.

## Table S5 – Sex-stratified analyses: males absolute changes from baseline

|  | **Low Load** | | | **High Load** | | | **P-value (Mann- Whitney U)** |
| --- | --- | --- | --- | --- | --- | --- | --- |
|  | **Median** | **IQR** | **n** | **Median** | **IQR** | **n** |  |
|  |  |  |  |  |  |  |  |
| HR, 24h | 1 | 6 | 13 | 2 | 9 | 10 | >0.999 |
| HR, Morning | 0.1 | 7 | 13 | 1 | 8 | 10 | 0.773 |
| HR, Afternoon | 0.0 | 20 | 13 | 2 | 32 | 10 | 0.693 |
| HR, Evening | 0.3 | 13 | 13 | -1 | 20 | 9 | >0.999 |
| HR, Night | 1 | 6 | 13 | -1 | 12 | 9 | 0.086 |
| HRR | -0.1 | 7 | 13 | -3 | 7 | 10 | 0.521 |
| Relative HRR (%HRR) | -1.0% | 21.4% | 13 | 3.8% | 32.5% | 10 | 0.483 |
| Systolic Blood Pressure | -5 | 13 | 13 | -6 | 13 | 11 | 0.539 |
| Diastolic Blood Pressure | -2 | 15 | 13 | 0.0 | 10 | 11 | 0.431 |
|  |  |  |  |  |  |  |  |
| **Time Domain (ms)** |  |  |  |  |  |  |  |
| SDNN, 24h | 2 | 36 | 13 | 18 | 32 | 10 | 0.088 |
| SDNN, Morning | **32 *** | 33 | 13 | 7 | 46 | 10 | 0.376 |
| SDNN, Afternoon | 9 | 28 | 13 | -7 | 34 | 10 | **0.021** |
| SDNN, Evening | 11 | 47 | 13 | 0.4 | 37 | 9 | 0.123 |
| SDNN, Night | -12 | 34 | 13 | -4 | 29 | 9 | 0.471 |
|  |  |  |  |  |  |  |  |
| RMSSD, 24h | 0.2 | 3 | 13 | 2 | 6 | 10 | 0.250 |
| RMSSD, Morning | 1 | 8 | 13 | 1 | 19 | 10 | 0.879 |
| RMSSD, Afternoon | 0.4 | 9 | 13 | -2 | 20 | 10 | 0.091 |
| RMSSD, Evening | -0.1 | 8 | 13 | -3 | 12 | 9 | 0.255 |
| RMSSD, Night | -3 | 15 | 13 | 1 | 12 | 9 | 0.647 |
|  |  |  |  |  |  |  |  |
| **Frequency Domain** |  |  |  |  |  |  |  |
| **Normalized values (n.u.; ms^2^)** |  |  |  |  |  |  |  |
| P_HF_ n.u., morning | -5 | 17 | 12 | 2 | 12 | 11 | 0.211 |
| P_HF_ n.u., afternoon | 3 | 12 | 12 | 1 | 16 | 10 | 0.771 |
| P_HF_ n.u., evening | 3 | 8 | 12 | 0.1 | 8 | 10 | 0.381 |
| P_HF_ n.u., night | 2 | 14 | 12 | -5 | 14 | 9 | 0.193 |
|  |  |  |  |  |  |  |  |
| P_LF_ n.u., morning | 5 | 19 | 12 | -2 | 12 | 11 | 0.260 |
| P_LF_ n.u., afternoon | -4 | 10 | 12 | -1 | 16 | 10 | 0.821 |
| P_LF_ n.u., evening | -3 | 8 | 12 | -0.3 | 9 | 10 | 0.314 |
| P_LF_ n.u., night | -3 | 11 | 12 | 0.1 | 15 | 9 | 0.193 |
|  |  |  |  |  |  |  |  |
| Ratio P_LF_/P_HF_ n.u., morning | 0.5 | 2.1 | 12 | -0.6 | 3.7 | 11 | 0.235 |
| Ratio P_LF_/P_HF_ n.u., afternoon | -1.4 | 3.5 | 12 | -0.2 | 8.7 | 10 | 0.674 |
| Ratio P_LF_/P_HF_ n.u., evening | -1.0 | 2.4 | 12 | -0.3 | 2.6 | 10 | 0.539 |
| Ratio P_LF_/P_HF_ n.u., night | -0.1 | 1.4 | 12 | 0.5 | 2.7 | 9 | 0.651 |

**Table S5. Absolute change from baseline in cardiovascular and HRV parameters among males.** Values are medians with interquartile ranges (IQR) for all males who completed the protocol. HRV parameters were derived from 24-hour ECG recordings, and blood pressure measured in the morning. Time- and frequency domain measures were analyzed over predefined 1-hour periods (morning, afternoon, evening, night). Statistically significant within-group changes are shown in bold. **P < 0.05.*

**Abbreviations:** are provided as shown in Table 1.
